# MMTR/Dmap1 Sets the Stage for Early Lineage Commitment of Embryonic Stem Cells by Crosstalk with PcG Proteins

**DOI:** 10.3390/cells9051190

**Published:** 2020-05-11

**Authors:** Young Jin Lee, Seung Han Son, Chang Su Lim, Min Young Kim, Si Woo Lee, Sangwon Lee, Jinseon Jeon, Dae Hyun Ha, Na Rae Jung, Su Youne Han, Byung-Rok Do, Insung Na, Vladimir N. Uversky, Chul Geun Kim

**Affiliations:** 1 Institute of Pharmaceutical Science and Technology, Hanyang University, Ansan, Gyeonggi-do 15588, Korea; 2 Department of Life Science and Research Institute for Natural Sciences, College of Natural Sciences, Hanyang University, Seoul 04763, Korea; imsangok12@naver.com (S.H.S.); sdpilot@hanmail.net (C.S.L.); 5718my@naver.com (M.Y.K.); wlsdk9727@naver.com (S.W.L.); lsw1346@naver.com (S.L.); 975870@naver.com (J.J.); dhha0719@gmail.com (D.H.H.); seint3725@nate.com (N.R.J.); hsy1232@hanmail.net (S.Y.H.); nainsung@hanyang.ac.kr (I.N.); 3 Biotechnology Research Institute, Hurim BioCell Inc, Seoul 07531, Korea; brokdo@hurimbiocell.com; 4 Department of Molecular Medicine, Morsani College of Medicine, University of South Florida, Tampa, FL 33612, USA; vuversky@usf.edu; 5 Institute for Biological Instrumentation of the Russian Academy of Sciences, 142290 Pushchino, Russia

**Keywords:** MMTR/Dmap1, Tip-p400 complex, polycomb repressive complexes (PRCs), bivalency, poised gene, embryonic stem cells

## Abstract

Chromatin remodeling, including histone modification, chromatin (un)folding, and nucleosome remodeling, is a significant transcriptional regulation mechanism. By these epigenetic modifications, transcription factors and their regulators are recruited to the promoters of target genes, and thus gene expression is controlled through either transcriptional activation or repression. The *Mat1*-mediated transcriptional repressor (MMTR)/DNA methyltransferase 1 (DNMT1)-associated protein (Dmap1) is a transcription corepressor involved in chromatin remodeling, cell cycle regulation, DNA double-strand break repair, and tumor suppression. The Tip60-p400 complex proteins, including MMTR/Dmap1, interact with the oncogene Myc in embryonic stem cells (ESCs). These proteins interplay with the stem cell-related proteome networks and regulate gene expressions. However, the detailed mechanisms of their functions are unknown. Here, we show that MMTR/Dmap1, along with other Tip60-p400 complex proteins, bind the promoters of differentiation commitment genes in mouse ESCs. Hence, MMTR/Dmap1 controls gene expression alterations during differentiation. Furthermore, we propose a novel mechanism of MMTR/Dmap1 function in early stage lineage commitment of mouse ESCs by crosstalk with the polycomb group (PcG) proteins. The complex controls histone mark bivalency and transcriptional poising of commitment genes. Taken together, our comprehensive findings will help better understand the MMTR/Dmap1-mediated transcriptional regulation in ESCs and other cell types.

## 1. Introduction

Preimplantation embryo-derived embryonic stem cells (ESCs) are able to self-renew and differentiate into multi-lineage cell types in vitro and in vivo with a normal karyotype [1,2,3]. Due to the fact of their contributions to the normal tissue or organ development after in vitro differentiation, ESCs have a promising potential for cell therapeutic development in regenerative medicine which defines the tremendous public attention to these cells [4,5]. To expand the range of cell types derived efficiently from the in vitro differentiation and thereby broaden the successful use of ESC-derived cells in medical applications, good understanding of the molecular mechanisms underlying self-renewal and pluripotency of ESCs is needed. However, this important research area is not fully elucidated yet. Several genes, including *Sox2*, *Oct4*, *Klf4*, and *Nanog* have been identified as crucial factors for gene expression control, forming a core pluripotency network in collaboration with transcriptional co-regulators [6,7,8,9]. Rapid alternations in expression of these genes during differentiation are connected with the bivalent chromatin structure changes, thereby affecting functions of corresponding promoters [10,11,12]. Importantly, it has been proposed that the overall ESC specific gene expression signature is composed of small sets of modules such as the core pluripotency factors (Core module), the Polycomb complex factors (PRC module), and the Myc-related factors (Myc module) [13]. The Core module is composed of genes co-occupied by at least seven out of nine factors of the Core cluster (Smad1, Stat3, Klf4, Oct4, Nanog, Sox2, Nac1, Zfp281, and Dax1), whereas the PRC module genes are the common targets of Polycomb group (PcG) proteins such as Suz12, Eed, Phc1, and Rnf2. The Myc module is composed of genes that are common targets of seven factors (Myc, Max, nMyc, E2F1, E2F4, and Zfx) in the Myc cluster. Although approximately one-third of all active ESC genes are bound by both c-Myc and the core ESC pluripotency factors [14], the Core and Myc-centered subnetworks in ES cells are separable units with unique roles in maintaining ES cell self-renewal [13]. In fact, the Core ESC factors select ESC genes for expression through the recruitment of RNA Pol II, whereas c-Myc functions to control gene expression through the release of transcriptional pause [15,16]. However, there is a lack of knowledge about how these modules crosstalk with each other to control the stemness and/or pluripotency of ESCs at molecular and cellular levels, although there has been a plethora of genome-wide transcriptional network data.

The DNA methyltransferase 1-associated protein (Dmap1) was originally identified as a protein associated with DNA methyltransferase 1 (Dnmt1) and is implicated in gene regulation through chromatin modification [17]. In addition, the Dmap1–Dnmt1 and the p33ING1-Sin3-histone deacetylase (HDAC) complexes bind pericentric heterochromatin. These two complexes are known to maintain the heterochromatin structure and histone modification in the late S phase [18]. Both Dmap1 and Dnmt1 colocalize throughout the S phase in somatic cells in order to mediate transcription repression. They also form DNA replication foci with HDAC2 during the late S phase in order to construct transcription repressive chromatin. Dmap1 is also a core component of the Tip60-p400 histone acetyltransferase (HAT) complex (or NuA4 HAT complex) and the ATP-dependent chromatin-remodeling complex Swr1/SRCAP [19,20,21,22,23]. In addition, Dmap1 is involved in the DNA double-strand break repair [24] and tumor suppression [25]. Recently, Kokosar and colleagues [26] reported that Dmap1 was heterogeneously expressed in adipose tissue in women with polycystic ovary syndrome (PCOS), which resulted in the epigenetic and transcriptional alternations. Despite all these observations, the exact roles of Dmap1 in cellular functions remain largely unknown.

Earlier, we characterized the MAT1-mediated transcriptional repressor (MMTR) from mouse ESCs as a novel clone and found it to be identical to Dmap1 [27]. MMTR is a key component of the RNA Pol II-mediated gene expression that interacts with HDAC1, and it modulates transcription factor IIH (TFIIH) kinase activity via MAT1 interaction [28,29]. We showed that the coiled–coil domain at the middle of MAT1 interacts with the C-terminal half of MMTR and that the MMTR-mediated transcriptional repression can be completely restored by the MAT1 overexpression in the presence of the HDAC1 inhibitor, trichostatin A (TSA). MMTR inhibited in vitro phosphorylation of the TFIIH kinase substrate, the C-terminal domain of the largest subunit of RNA Pol II. This mechanism is important for efficient promoter escape via early termination of Pol II elongation [30]. We also found that MMTR is an intrinsic negative cell cycle control factor that modulates cyclin-dependent kinase (Cdk)-activating kinase (CAK) kinase activity via an interaction with MAT1 [28,29]. CAK (composed of the catalytic subunit Cdk7, the regulatory subunit cyclin H, and MAT1) is a sub-complex of TFIIH [31] and preferentially phosphorylates Cdks to induce G1/S and G2/M phase transitions.

In terms of the ESC physiology, MMTR/Dmap1 is critical for pluripotency as a subunit of the Tip60-p400 complex [32]. The homozygous knock-out mice died prior to implantation (examined as early as the 8 cell embryo stage) [33]. Importantly, Tip60-p400 complex proteins interact with the oncogene Myc in ESCs. The proteins of this complex are involved in the network and regulate expression levels of various genes, potentially through the histone-acetyltransferase and/or the H2A.Z-exchanging activities of the complex [13]. As such, the roles of MMTR/Dmap1 in ESCs have not been fully understood. Here, we investigated the functions of MMTR/Dmap1 in mouse ESCs (mESCs). We analyzed MMTR/Dmap1 target genes that were preferentially expressed during both maintenance and differentiation of mESCs. To this end, we performed a systemic analysis of MMTR/Dmap1 binding, chromatin organization, and transcriptional programs within the mESCs. Furthermore, MMTR/Dmap1 programs were compared with already known DNA methylation and histone acetylation functions to determine the roles of this protein in the regulation of poised target genes. These genes are tightly controlled during maintenance or spontaneous differentiation of mESCs. MMTR/Dmap1 was more active within the Tip60-p400 complex to maintain the bivalent histone marks via the competitive binding to Polycomb repressive complexes (PRCs) at the promoter. Furthermore, the abnormal level of MMTR/Dmap1 and its deletion mutants (N- or C-terminal fragment of MMTR/Dmap1) produced by ectopic overexpression or shRNA-mediated knockdown of MMTR/Dmap1 caused differentiation failure due to the Tip60-p400 complex’s destabilization. Our studies demonstrate novel functions of MMTR/Dmap1 in mESCs maintenance and differentiation at histone acetylated target genes rather than methylated genes by its binding to Dnmt1.

## 2. Materials and Methods

### 2.1. Cell Culture

The HEK (Human Embryonic Kidney) 293T-17 cells were cultured in Dulbecco’s modified Eagle’s medium (DMEM; Hyclone, South Logan, UT, USA) with 10% fetal bovine serum (FBS; Hyclone), 50 units/mL penicillin (Sigma Aldrich, Saint Louis, MO, USA), and 50 μg/mL streptomycin (Sigma Aldrich) at 37 °C with 5% CO_2_. We cultured the mESC lines CCE (129/sv) and J1 (129S4/SvJae) under feeder-free conditions and maintained in DMEM with 15% heat-inactivated FBS, 1X MEM Non-Essential Amino Acids Solution (Sigma Aldrich), 300 μM monothioglycerol (Sigma Aldrich), 1X penicillin/streptomycin, and 1000 U/mL LIF (leukemia inhibitory factor, Sigma Aldrich) in 0.1% gelatin-coated cell culture dishes at 37 °C with 5% CO_2_. For differentiation, a hanging drop method [34] was employed. Cell aggregates were transferred to bacteria culture dishes on day 2 and further cultured in ESC medium without LIF. We monitored stemness by alkaline phosphatase staining with the Leukocyte Alkaline Phosphatase Assay Kit (Sigma Aldrich).

### 2.2. Establishment of MMTR/Dmap1 Full-Length, N-terminal, and C-terminal Half Overexpression and Knockdown ESC Lines

We constructed the pcDNA3 Flag-MMTR plasmid and the pEF1-3xFB-MMTR plasmid swapping the FlagBio sequences in the pEF1α-FlagBio-Dmap1 with 3xFLAG-Bio Sequences [28]. To generate the MMTR/Dmap1 shRNA expression vector, complementary oligonucleotide sequences (corresponding to +510 to +528 nucleotides of mouse MMTR/Dmap1 from the transcription start site) were synthesized, annealed to form dsDNA, and subcloned into the pSuper-Retro vector cut with BamHI and BglII. Using shRNA oligonucleotides with 2 base mismatches, we prepared the MMTR/Dmap1 mutant shRNA (mshRNA) plasmid. The EcoRI/ClaI fragment from the pSuper-Retro-MMTR/Dmap1 shRNA and mshRNA was inserted into the EcoRI/ClaI cut pLV-TH to generate pLV-TH-MMTR/Dmap1 shRNA and mshRNA plasmids, respectively. The MMTR N-terminal (1–220) and C-terminal (221–468) fragments were amplified through PCR of pcDNA3-Flag-MMTR. Then, corresponding PCR products were cloned into the pcDNA3 Flag vector using BamHI. Primer sequences for PCR reaction were as follows: MMTR-N-BamHI (F); GGA TCC TA A TGG CTA CGG GC, MMTR-N-BamHI (R); GGA TCC AGC ATC AAA CAC TGG, MMTR-C-BamHI (F); GGA TCC GCG GGC ATG AGA GAC GG, MMTR-C-BamHI (R); GGA TCC TGG TTT CTT GGC TTT C. The ESC lines overexpressing MMTR full-length, N- and C-terminal fragments were obtained by pcDNA-Flag-MMTR/Dmap1 transfection into ESCs using Lipofectamine 2000 reagent (Invitrogen, Waltham, MA, USA). We selected cell lines with 1 μg/mL puromycin (Sigma Aldrich). In the meantime, cotransfection of the pLV-TH-MMTR/Dmap1 shRNA into HEK 293T cells with pMD.G and pΔ8.2 [35] was conducted to generate lentiviruses containing pLV-TH-MMTR/Dmap1 shRNA. Finally, MMTR/Dmap1 shRNA ESC lines were established by prepared lentivirus transfection into ESCs in the presence of 8 μg/mL polybrene (Sigma Aldrich). We isolated EGFP expressing cell clones under a fluorescent microscope. We confirmed nucleotide sequences and plasmid expression by DNA sequencing and Western blots on the cell extracts obtained from the transfected 293T cells, respectively.

### 2.3. Total RNA Preparation and RT-qPCR

Total cellular RNA was extracted using Qiazol reagent (Qiagen, San Diego, CA, USA) according to the manufacturer’s procedures for RT-PCR. Purified RNA was resuspended in DEPC water. Four hundred nanograms of total RNA were used for cDNA synthesis using 10 pmol random hexamer and the High Capacity cDNA Reverse Transcription Kit (Toyobo, Osaka, Japan). We conducted RT-qPCR using SYBR green (TaKaRa, Kusatsu, Japan) with the specific primers (Supplementary Materials Table S1). Amplifications were performed using a light cycler 1.5 real-time PCR system (Roche, Basel, Switzerland). Transcript quantification was calculated as 2^−ΔCt^, based on ΔCt = ΔCt (treated) − ΔCt (untreated), with GAPDH levels as internal controls. Errors were calculated from at least two independent experiments.

### 2.4. Western Blot

Western blots were performed as previously described [36]. Proteins in cell lysates were resolved by electrophoresis on 6%–10% polyacrylamide gels and subsequently transferred to PVDF membranes (GE-Healthcare). Proteins were visualized by chemiluminescence using an ECL system (GE Healthcare).

### 2.5. Cell Cycle Analyses Using Flow Cytometry

Cells were trypsinized, washed in 0.1% BSA in PBS, counted, and resuspended in 2% FBS/0.01% NaN_3_ at 1 × 10^6^ cells/100 μL. Cells were washed, fixed with 10% formalin in PBS, stained with 5 mg/mL propidium iodide (Sigma Aldrich) with 10 mg/mL RNase A for 10 min, and analyzed using FACSCalibur (BD Biosciences, San Jose, CA, USA).

### 2.6. In Vivo Differentiation Assay

Immunodeficient SCID mice (ICN nude) were used for the teratoma assay. Cells from each mESCs line were mechanically detached from the surface. Obtained cell aggregates (5 × 10^6^ cells) were injected with sterile 26 G needle subcutaneously into the dorsoposterior region of 7~8 week old mice. Teratomas were obtained at 4 weeks post-injection. All animal procedures were approved by the Animal Care and Use Committee and the Institutional Review Board of Hanyang University

### 2.7. Chromatin Immunoprecipitation-Quantitative PCR (ChIP-qPCR) Analysis

The ChIP-qPCR was performed as described [37], using the appropriate ChIP-grade antibodies (H3K27me3, Cat#ab6002; H3K27ac, Cat#ab4729; Abcam, Cambridge, MA, US; mouse IgG, Cat#31430; Thermo Fisher, Waltham, MA, USA). Then, we performed RT-qPCR using SYBR green (TaKaRa) and the specific primers (Table S1). The data were normalized to the input DNA and enrichment was calculated by fold excess over ChIP performed with specific IgG.

### 2.8. Microarray Data Analysis

Total RNA was extracted using Trizol (Invitrogen) and purified using RNeasy columns (Qiagen) according to the manufacturer’s protocol. After DNase digestion and clean-up procedures, RNA samples were quantified, aliquoted, and stored at −80 °C. For quality control, RNA purity and integrity were analyzed on Agilent 2100 Bioanalyzer (Agilent Technologies). Total RNA was amplified and purified using the Ambion Illumina RNA amplification kit (Ambion, Auatin, TX, USA) to yield biotinylated cRNA according to the manufacturer’s instructions. Seven hundred and fifty nanograms of labeled cRNA samples were hybridized to each Mouse WG-6 expression v.2 bead array for 16–18 h at 58 °C, according to the manufacturer’s instructions (Illumina, San Diego, CA, USA). Array signal was detected using Amersham fluorolink streptavidin-Cy3 (Invitrogen) following the bead array manual. The quality of hybridization and overall ChIP were monitored by visual inspection of both internal quality control checks and the raw data. Raw data were obtained using the software, Illumina GenomeStudio v2011.1 (Gene Expression Module v1.9.0). Array probes with detection *p*-value > 0.05 (signal to noise) in over 50% samples were filtered out. Selected gene signal value was transformed by logarithm and normalized by quantile method. Raw Microarray data were analyzed at October, 2013 and uplosded at February, 2020 through the Gene Expression Omnibus under accession no. GSE145561 [38]. Data post-processing and graphics were performed with in-house developed functions in MATLAB. Hierarchical clusters of genes and samples were performed with a one minus correlation metric and the average (unweighted pair group) linkage method. Official gene symbols were annotated with the MGI 2015 July database. The change in the RNA expression profile during mESC differentiation (d0 and d3) were compared with the change in the RNA expression profile of hESC differentiation (d0 and d12) from GSE15257.

### 2.9. ChIP Data Processing and Analyses

We used the following Public ChIP-data sources (GEO Datasets): DNA methylation (GSM1341316, GSM1656422, GSM1656423), Dmap1 (GSM1183112), Tip60 (GSM1183114), p400 (GSM1269901), Max (GSM1183115), Myc (GSM1183111), Suz12 (GSM1041374), Cbx7 (GSM1041373), Ring1B (GSM1041372), Ezh2 (GSM590132), H3K4me3 (GSM769008), H3K27ac (GSM1000099), H3K27me3 (GSM1000089), and H2A.Z (GSM849928). Each fastq file was converted into fastqsanger format using the Fastqgroomer utility (Galaxy version 1.0.4; https://usegalaxy.org/), and the resulting files were mapped with mouse reference genome mm9 using the Bowtie2 (Galaxy Version 2.2.6) utility. Peak calling was performed by MACS (Galaxy Version 1.0.1), and each binding peak’s gene was annotated by the BETA-minus tool in Galaxy cistrome (http://cistrome.org/ap/root#) using the <20 kb of each peak and <100 kb of TSS option. Binding regions were called by CEAS (Enrichment on chromosome and annotation, Ver 1.0.0) in Galaxy cistrome. Common factor binding regions were analyzed by intersect tools (Galaxy Version 1.0.0). Heatmap analyses were performed using the R-bioconductors package in Galaxy cistrome version 1.0.0. De novo DNA binding motif search was performed using MDscan algorithm, and similar DNA binding motif identification was performed using cistrome motif database in Galaxy cistrome SeqPos motif tool [39].

### 2.10. Bioinformatics Analysis

Enrichment term analyses for the microarray and ChIP-seq data were performed using the Clue GO program (v. 2.1.7) and Cytoscape 3.0 (v.3.2.1). To identify the binding position of the target peak in the UCSC genome browser (http://www.genome.ucsc.edu/), the wig format was converted to the bigwig format. The stemness and differentiation-related enrichment was analyzed using the GSEA2 utility (http://software.broadinstitute.org/gsea/index.jsp) (v.2.2.0) with the 2015 July database. Data are presented as the mean +/− standard error. The sample size for each experiment, *n*, is included in the results section (Section 3) and the associated figure legends. The differences between two subsets of data are considered statistically significant if the one-tailed Student’s *t*-test gives a significance level *p* (*p*-value) less than 0.05. Statistical analyses were performed using IBM SPSS statistics 23.

## 3. Results

### 3.1. MMTR/Dmap1 Is Required for Accurate Differentiation of mESCs

**Figure 1:**
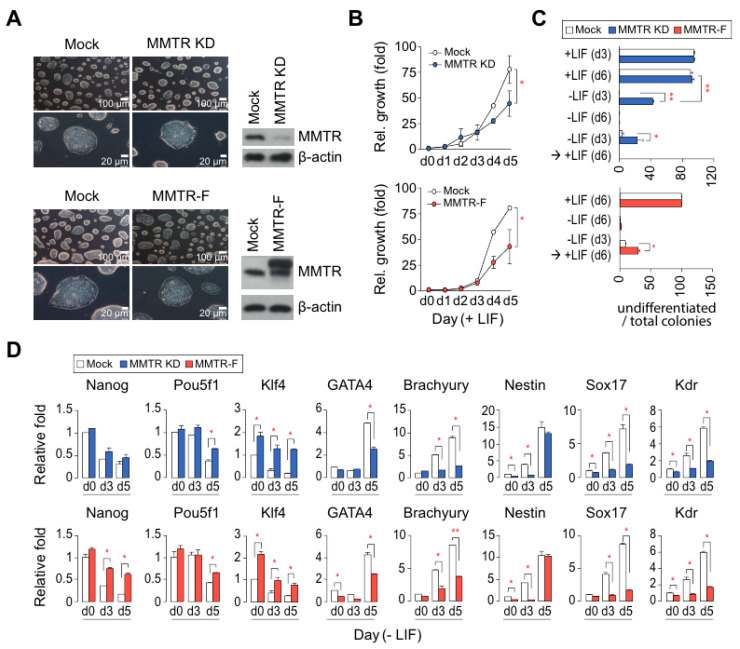
Establishment and characterization of *Mat1*-mediated transcriptional repressor (MMTR) KD and MMTR–F mESC lines. (**A**) MMTR KD and MMTR-F clones maintain typical morphologies under phase contrast microscope. (**B**) Cell proliferation rates for 5 days under LIF. (**C**) Differential potentials of MMTR KD (left) or MMTR-F (right) cells during 6 days in the present or absence of LIF measured by cell colonies with AP staining. We scored numbers of AP positive colonies (undifferentiated colonies) out of total colonies. (**D**) RT-qPCR of stemness and three germ layer specific markers. *n* = 2. * *p* < 0.05; ** *p* < 0.01. KD, knockdown; F, full-length; LIF, leukemia inhibitory factor; AP, alkaline phosphatase, RT-qPCR, reverse transcription-quantitative polymerase chain reaction.

We established MMTR knockdown (MMTR KD) or MMTR/Dmap1 full-length overexpressed (MMTR-F) mESCs to test whether MMTR/Dmap1 is crucial for the maintenance and/or differentiation of mESCs. Morphologies of the established cell lines were not significantly different from each other (Figure 1A). Meanwhile, the cell proliferation rates of both mESCs were reduced (Figure 1B). From flow cytometry, we observed slight changes in cell cycle distribution from both MMTR KD and MMTR-F mESCs: G2/M increase and G0/G1 decrease (Figure S1A). Interestingly, the population level of undifferentiated cells in both MMTR KD and MMTR-F mESCs was higher than that of controls under the differentiating condition (−LIF) or 3 day culture in the absence of LIF followed by an additional 3 day culture in the presence of LIF (Figure 1C). Both MMTR KD and MMTR-F showed small embryoid bodies and/or dissociation of cells leading to cell death during differentiation in the absence of LIF for 5 or 6 days. In addition, expression levels of pluripotency markers (Nanog, Pou5f1, and Klf4) were mostly sustained, while those of three germ-layer markers (GATA4, Brachyury, Nestin, Sox17, and Kdr) were delayed during differentiation for 5 days (Figure 1D). Consistent with gene expression profiles, differentiation potential in vivo, as revealed by teratoma forming ability, appeared in control mock but not in MMTR-F or MMTR KD mESCs-injected mice (Figure S1B). These findings support the hypothesis that MMTR/Dmap1 plays a critical role in the regulation of the commitment of differentiation but does not have noticeable effects on the maintenance of mESCs self-renewal.

### 3.2. The Distinct Roles between N- and C-Terminal Regions of MMTR/Dmap1 in Differentiation of mESCs

**Figure 2:**
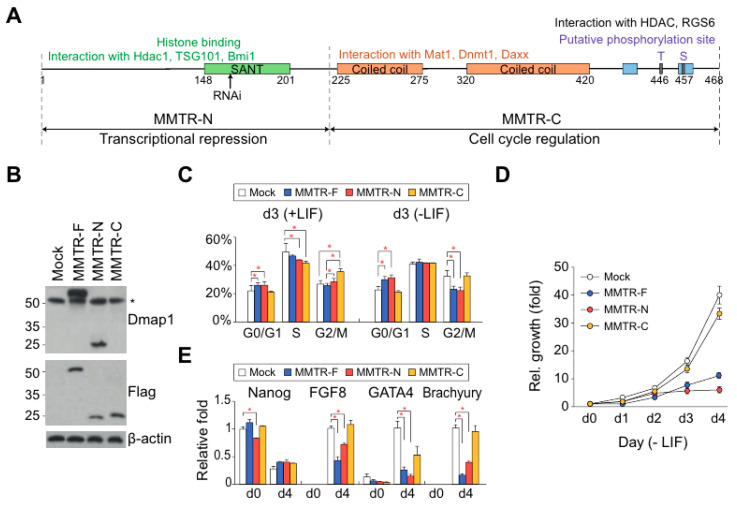
Establishment and characterization of MMTR-F, -N, and -C mESC lines. (**A**) Diagram of each MMTR mutant clone and (**B**) validation of their expressions by immunoblotting. Specific antibodies for immunoblots are shown at the right side of the photos, and the asterisk (*) denotes endogenous MMTR/Dmap1. (**C**) Cell cycle profile by flow cytometry. (**D**) Cell proliferation rates. (**E**) RT-qPCR of stemness and three germ layer specific markers. *n* = 2. * *p* < 0.05.

To test the functional roles of MMTR/Dmap1 domains, N- and C-terminal mutants (MMTR-N and MMTR-C, respectively) were constructed and overexpressed in mESCs (Figure 2A,B). MMTR-F and MMTR-N cells showed similar cell cycle distributions under both undifferentiated and differentiated conditions compared to those of the wild-type mESCs (Figure 2C). In addition, MMTR-C and wild-type mESCs cell cycle distributions were also not distinguishable under both culture conditions (Figure 2C). Interestingly, the MMTR-F and MMTR-N mESCs showed lower cell proliferation rates than wild-type or MMTR-C mESCs (Figure 2D and Figure S1C,D). The expression levels of pluripotency markers (e.g., Nanog) was reduced only in MMTR-N mESCs from d0 to d4, while differentiation markers (FGF8, GATA4, and Brachyury) expression levels increased later in MMTR-F and MMTR-N mESCs (Figure 2E). The contribution of target gene regulation activity of MMTR-N was also very similar to that of the MMTR-F [40] (Figure S2A,B). In addition, teratoma lesions appeared in mock and MMTR-C but not MMTR-F or MMTR-N mESCs-injected mice (Figure S1E). These data suggest that the N-terminal region of MMTR/Dmap1 possesses target genes expression stimulation activity with partner chromatin remodeling complexes. However, we noted that the C-terminal half of MMTR is involved in limited functions of histone protein synthesis and cell cycle regulation during differentiation (Figure S2C,D), although MMTR-C mESCs have a similar decrease to control mock cells in gene expression of differentiation markers and cell proliferation rate (Figure 2C,D). Taken together, deregulation of MMTR/Dmap1 (by MMTR-F, -N, -C, and KD) leads to the developmental disorder due to the abnormal onset of differentiation. It supports our interpretation that MMTR/Dmap1 serves as a molecular fate decision rheostat of mESCs.

### 3.3. MMTR/Dmap1 Functions as a Member of the Tip60-p400 Complex for Chromatin Remodeling during Commitment of mESC Early Differentiation

Taking into account that the early differentiation of mammalian ESCs mimics early embryo development [41], we examined microarray-based gene expression profiles of MMTR-F, -N, -C, and KD cells at the undifferentiated (d0) and differentiated states (d3). During differentiation of mESCs, expression of diverse genes was observed, as it was shown previously [42]. In fact, all MMTR mutant cells did not differentiate in their expression on day 0, but all mutants differentiated on day 3 (Figure S3A and B). Three days of differentiation-affected gene expression profile, but MMTR gene mutations did not cause noticeable differences (Figure S3A). For instance, we found that gene expression profiles of WT and MMTR-F were like each other at d0 or d3, but more different gene expression profiles appeared in d0 and d3 of WT mESCs (Figure S3B). We found that 2104 genes (7.8%) out of 26,955 genes were significantly changed in the array when comparing d3 to d0 in WT mESCs (|log_2_ Fc| ≥ 0.5). The significantly changed genes comprised 1084 upregulated genes (4.02%) and 1020 downregulated genes (3.78%) (Figure S3C). Preferentially expressed genes in each of the MMTR mutants on day 0 and day 3 were not abundant (Figure S3D), and we confirmed their expression by RT-qPCR (Figure S3E). When we analyzed the dataset by principal component analysis (PCA) to identify distinctive clusters based on the gene variability among the MMTR mutants between day 0 and day 3, three molecular subtypes at day 3 (MMTR-N/MMTR-F, MMTR-C/WT, and MMTR KD) were distinguishable from day 0 (Figure S3F). It was also notable that the MMTR KD cluster at day 0 was distinguishable from other groups.

**Figure 3:**
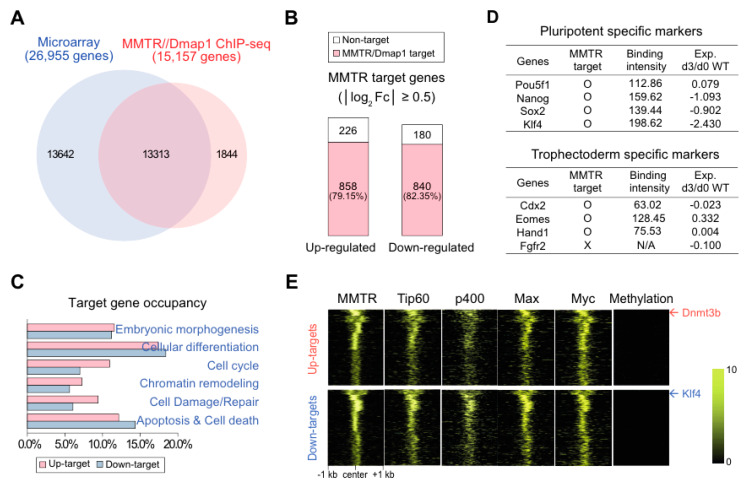
Alteration of MMTR/Dmap1 target gene expression during spontaneous mESCs differentiation. (**A**) Venn diagram of overlapping gene numbers at day 0 WT mESCs in the microarray and genes targeted by MMTR/Dmap1 in CHIP-seq (GSM1183112, TSS ≤ ± 20 kb). We indicated the number of overlapping and non-overlapping genes. (**B**) Percentage of MMTR/Dmap1 target genes among those with significant (|log_2_ Fc| ≥ 0.5) up- or downregulation during spontaneous mESCs differentiation. (**C**) The mESC differentiation commitment-related gene ontology (GO) biological process in up- or downregulated MMTR/Dmap1 targets. (**D**) Dependency of expression change to pluripotency and trophectoderm targets of MMTR/Dmap1 during mESC differentiation. MMTR target = Binding intensity ≥ 60; Binding intensity = −log_10_ (*p*-value); Expression changes = log_2_ fold change. (**E**) ChIP-seq heat maps showing occupancies of MMTR/Dmap1, Tip60, p400, Max, Myc, and the DNA methylation status in the promoters of MMTR/Dmap1 up- and down-target genes in undifferentiated mESCs. Dnmt3b and Klf4 are representative for MMTR/Dmap1 up- or downtarget genes, respectively. The color bar indicates binding strength. GO, gene ontology.

Only 49.3% (13,313 of 26,955) genes in the microarray were binding targets of MMTR/Dmap1 by public ChIP-Seq data (GSM1183112, TSS ≤ ± 20 kb) analyses (Figure 3A). Interestingly, among 2104 genes significantly (|log2 Fc| ≥ 0.5) changed in their expression during mESCs differentiation, 858 (79.15%) and 840 (82.35%) genes were identified as up- or downregulated MMTR/Dmap1 targets, respectively (Figure 3B), suggesting that the majority of differentially expressed genes during commitment of mESC differentiation were regulated by MMTR/Dmap1. As expected, gene ontology (GO) analysis showed that these MMTR/Dmap1 target genes are categorized into highly significant terms of embryo (or cell) development, embryonic morphogenesis, cellular differentiation, cell cycle, chromatin remodeling, cell damage/repair, and apoptosis/cell death (Figure 3C). At this point, we analyzed the gene expression profiles of MMTR/Dmap1-target pluripotency (*Pou5f1*, *Nanog*, *Sox2*, and *Klf4*) and trophectoderm (*Cdx2*, *Eomes2*, *Hand1*, and *Fgfr2*) markers to verify whether these genes influence the lineage commitment during mESCs differentiation. Although MMTR/Dmap1 binds to the regulatory region(s) of both pluripotency and trophectoderm markers, significant expression changes appeared only in the pluripotency markers during commitment of differentiation (Figure 3D and Figure S4). These findings suggest that not all MMTR/Dmap1 target genes change their expression during commitment of differentiation, and that in addition to the MMTR/Dmap1 binding, the expression fate of each MMTR/Dmap1 target in mESCs is context dependent.

MMTR/Dmap1 was initially identified to bind to Dnmt1 for DNA methylation [17,43]. The MMTR/Dmap1-containing complex is also a component of the Tip60-p400 complex [12,21,32]. To understand the interconnection between these two different phenomena in mESC commitment gene promoters, we analyzed public ChIP-seq database for other Tip60-p400 complex members, including Tip60, p400, Max, and Myc. As shown in Figure 3E and Figure S5A, almost all MMTR/Dmap1-bound regions showed strong association with Tip60, p400, Max, and Myc. However, these regions did not correlate with the CpG methylation status (Figure S5A). In addition, the MMTR/Dmap1-containing Tip60-p400 complex was rarely bound to the highly methylated CpG islands. Furthermore, we performed de novo motif analysis and cistrome analysis of TSS +/− 1 kb sequences of MMTR/Dmap1 up- and down-target genes to analyze motif(s) at MMTR/Dmap1 binding sites that overlapped with Myc and to check whether these motif(s) were Myc-related consensus sequences. As a result of integrating the analyses, we found that the Ets domain family and novel (CG-rich) consensus sequences were enriched in MMTR/Dmap1 binding sites (Figure S5B). In addition, 28% (477/1698) or 34% (579/1698) of MMTR/Dmap1 target genes were occupied or co-occupied by only MMTR/Dmap1 or MMTR/Dmap1-Myc, respectively (Figure S5C). Furthermore, 38% (642/1698) of target genes were occupied by MMTR/Dmap1 and Myc but not overlapped (Figure S5C). Then, Myc binding (Figure S5D) and MMTR/Dmap1-Myc common binding (Figure S5E) consensus sequences were identified from these analyses. Interestingly, we found that GC-rich motifs were enriched in MMTR/Dmap1-binding consensus sequences (Figure S5B,E,F). These observations suggested that newly identified CG-rich regions are important for the regulation of gene expression by MMTR/Dmap1-involved complexes.

Importantly, 15,157 raw peaks in the MMTR/Dmap1 ChIP-seq data met the peak-calling algorithm. These peaks were strikingly enriched around the transcriptional start site (TSS), such that approximately 40% of the promoter regions (between ± 3 kb) of target genes were occupied by MMTR/Dmap1, although 5’ UTR, intron, exon, and distal intergenic regions of targets showed MMTR/Dmap1 preference (Figure S5G). Based on the number of genes, their biological terms, and binding sites of the MMTR/Dmap1 targets, we hypothesize that the majority of genes that alter their expression by the mESC differentiation commitment (i.e., mESC commitment genes) are MMTR/Dmap1 target genes, and, therefore, MMTR/Dmap1 may play a critical role in the mESC commitment via the transcription control.

### 3.4. Suz12 Binding to the Commitment Gene Promoters, but not MMTR/Dmap1, Is Modulated Accompanied by H3K27 Modification during the mESCs Differentiation

**Figure 4:**
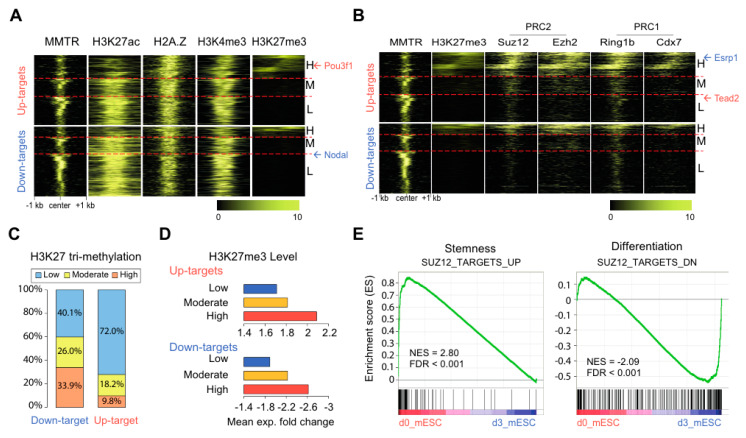
MMTR/Dmap1 co-localizes with PRC complex proteins at the promoters of the MMTR/Dmap1 up- and down-targets. (**A**) Heat maps showing histone modifications and H2A.Z at the MMTR/Dmap1 binding regions of up- and down-target promoters in undifferentiated mESCs. We sorted heat maps by the propensity of H3K27me3 modification (high, medium, and low levels). (**B**) Heat maps showing binding patterns of MMTR/Dmap1 and PRC proteins at the target promoters. The color bars indicate binding strength. (**C**) The proportion of H3K27me3 modification strengths in the MMTR/Dmap1 up- and down-targets. (**D**) The mean expression fold changes of both up- and down-targets during differentiation according to the strength of H3k27me3 modification. (**E**) GSEA2 analyses of MMTR/Dmap1 stemness (left) and differentiation (right) targets with differential expression by Suz12 knockout mESCs [53]. PRC, polycomb repressive complex; GSEA2, Gene Set Enrichment Analysis 2.

The Tip60-p400 complex activates gene expression through its histone acetyltransferase and ATP-dependent H2AZ-H2B dimer exchange activities [32,44]. We analyzed the presence of active (H3K4me3, H3K27ac) or inactive (H3K27me3) histone marks, and H2A.Z occupancy in the MMTR/Dmap1 binding regions of the mESC commitment genes. Based on heat map analysis, we found that (i) all active histone marks were occupied around the MMTR/Dmap1 binding center, and (ii) the strength of these active marks depended on the Tip60-p400 complex binding affinity (Figure 4A). Two distinct PRCs, PRC1 and PRC2, collaborate to set the stage for early lineage commitment by maintaining epigenetic repression of key developmental loci in ESCs [45], and this mechanism is conserved in eukaryotes [46,47]. PRC1 and PRC2 have histone modification activities, catalyzing mono-ubiquitination of histone H2A (H2AK119u1) and trimethylation of H3 lysine 27 (H3K27me3), respectively [48,49,50,51,52]. We tested co-occupancy of PcG proteins (Ring1b and Cbx7 of PRC1; Suz12 and Ezh2 of PRC2) and the Tip60-p400 complex at the mESC commitment gene promoters (up- and downregulated targets) in undifferentiated mESCs to examine MMTR/Dmap1 control activities (Figure 4B). As expected, those promoters with the increased binding of the PcG proteins showed a positive correlation with H3K27me3 marks but a negative correlation with the active histone marks and binding levels of Tip60-p400 complex. Conversely, those promoters with the increased binding of Tip60-p400 complex showed a positive correlation with the active histone marks but a negative correlation with the inactive H3K27me3 marks and binding levels of PcG proteins (Figure 4B).

Interestingly, the H3K27me3 modification strength was different between up- and downregulated genes during the mESC differentiation, such that 33.9% and 26.0% of the upregulated genes showed high or moderate H3K27me3 modification, respectively, whereas only 9.8% and 18.2% of the downregulated genes showed high or moderate H3K27me3 modification, respectively (Figure 4C). In both up- and downregulated MMTR/Dmap1 targets, genes marked with strong H3K27me3 modification tended to show higher expression level changes during the mESC differentiation (Figure 4D), suggesting that H3K27me3 actively controls the commitment of gene expression, whereas the Tip60-p400 complex passively controls the expression level during the mESC differentiation. Consequently, (i) genes actively transcribed but destined to be inactivated during differentiation contain strong active histone marks along with the weak or moderate H3K27me3, and (ii) genes in the poised state and to be activated for differentiation contain bivalent histone marks (H3K4me3 and H3K27me3) [11]. Based on these results, we propose that the interplay between the Tip60-p400 complex and the H3K27me3-modifying protein complex (i.e., PRC2) is crucial for mESC differentiation.

To further explore the relationship between the MMTR/Dmap1 and PRC complexes for gene regulation, we conducted Gene Set Enrichment Analysis 2 (GSEA2) [40] on gene sets that were differentially expressed in Suz12 knockout (KO) mESCs [53]. Polycomb protein Suz12 (suppressor of zeste 12 protein) is critical for H3K27 methylation as a docking factor of PRC2 [54]. Most of the upregulated genes in Suz12 KO cells (SUZ12_TARGET_UP) are involved in stemness, whereas most of the downregulated genes in Suz12 KO mESCs (SUZ12_TARGET_DN) are involved in differentiation (Figure 4E). Therefore, these data suggested that the interplay between MMTR/Dmap1-embeded Tip60-p400 and PRC complexes controls the mESCs commitment.

**Figure 5:**
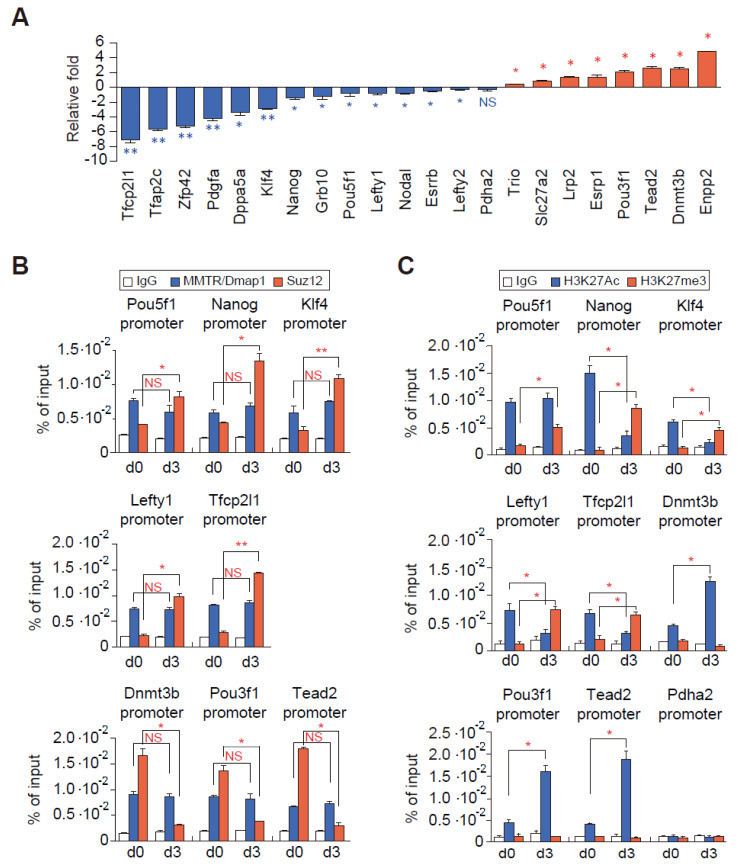
Modulation of Suz12 but not MMTR/Dmap1 binding to the commitment gene promoters occurs accompanying H3K27 modification during mESCs differentiation. (**A**) Bar graphs showing expression changes of representative MMTR/Dmap1 targets during mESC differentiation. Expression changes of these genes from the RT-qPCR data are in Figure S6A,B. Blue color bars indicate stemness genes, whereas red color bars indicate developmental genes. (**B**) ChIP-qPCR graphs showing MMTR/Dmap1 (blue) and Suz12 (red) bindings to the selected commitment gene promoters in undifferentiated (d0) and differentiated (d3) mESCs. Each ChIP value is presented as a percentage of the respective input DNA. (**C**) Quantification of H3K27 modification changes in the selected commitment gene promoters by ChIP-qPCR of undifferentiated mESCs (d0) and differentiated mESCs (d3). Pdha2 was a negative control. *n* = 2. * *p* < 0.05; ** *p* < 0.01; NS, no significant.

Some of the MMTR1/Dmap1 target genes are also regulated by Suz12 KO mESCs (Figure 4E), and therefore we tested both MMTR/Dmap1 and Suz12 occupancy and H3K27 modification status in selected target gene promoters (Figure 5). Expression of these selected genes was further confirmed by the RT-qPCR analysis (Figure 5A and Figure S6A,B). We found that the MMTR/Dmap1 occupancy to the promoter was not different between stemness and developmental genes and during differentiation (d0 versus d3), whereas the Suz12 occupancy showed an inverse correlation with the gene expression (i.e., high Suz12 binding was in d0 developmental genes and in d3 stemness genes) (Figure 5B). The H3K27me3 status was highly increased along with mostly decreasing of H3K27ac status in the stemness gene promoters during differentiation, whereas H3K27ac was highly increased along with no changes in H3K27 methylation status in developmental gene promoters (Figure 5C). These results suggested that the changes in expression of commitment genes during differentiation is regulated by the binding of Suz12 (or PRC complexes) but not by MMTR/Dmap1 (or Tip60-p400 complex) accompanying H3K27 modifications. Therefore, one can also suggest that the subtle interplay between the Tip60-p400 and PRC complexes regulates the MMTR/Dmap1 targeted commitment gene expressions during the maintenance and differentiation of mESCs.

### 3.5. PRC Complexes Function as an On–Off Switch, whereas the Tip60-p400 Complex Executes Gene Expression via Recruiting Factors Required for Gene Activation During the mESCs Differentiation

**Figure 6:**
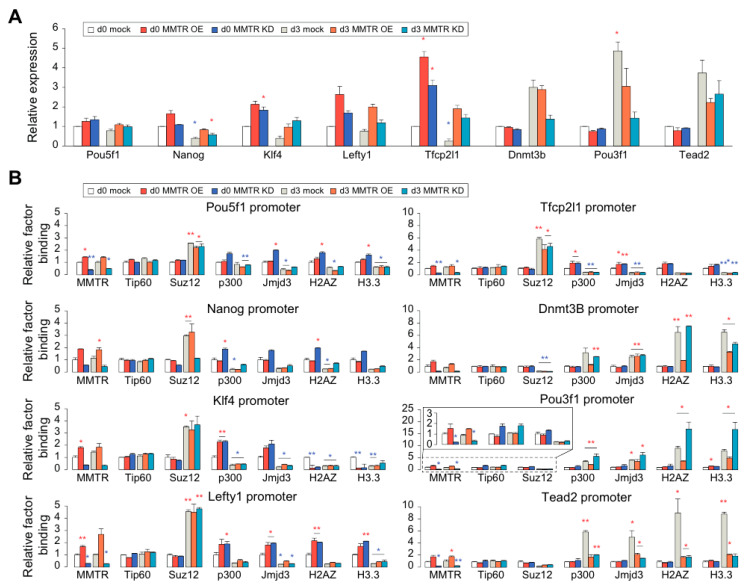
mESC commitment gene expression is differentially regulated by both MMTR/Dmap1 and Suz12 during mESCs differentiation. (**A**) RT-qPCR graphs showing selected mESC commitment genes expression in undifferentiated (d0) and differentiated (d3) MMTR OE and KD mESCs. We obtained expression levels of samples in each gene by RT-qPCR and depicted the relative folds to the d0 mock values. (**B**) ChIP-qPCR showing the relative binding of transcription control factors to the promoters of mESC commitment genes. Binding folds normalized by IgG values and relative folds were depicted to the d0 mock values. *n* = 2. * *p* < 0.05; ** *p* < 0.01.

To verify our hypothesis that interplay between the Tip60-p400 and PRC complexes regulates the MMTR/Dmap1 targets, we tested the correlation of the expression level with the MMTR/Dmap1 and Suz12 binding status in the promoters of selected commitment genes during differentiation of MMTR OE/KD mESCs (Figure 6). In general, both MMTR OE and KD induced higher expression of stemness-related MMTR/Dmap1 target genes in undifferentiated mESCs, when compared with the WT mock cells, whereas it did not affect expression of the development-related MMTR/Dmap1 target genes (Figure 6A), although MMTR OE and KD induced higher and lower MMTR binding to the commitment gene promoters, respectively (Figure 6B). Interestingly, another Tip60-p400 complex protein Tip60 did not change its binding affinity to the promoters by MMTR OE/KD (Figure 6B), suggesting that the MMTR deficient Tip60-p400 complex is still recruited to the promoters. On the contrary, Suz12 recruitment to the promoters was not affected by MMTR OE/KD in undifferentiated cells, whereas it was increased in the stemness gene promoters while decreased in the developmental gene promoters in differentiation day 3 (Figure 6B). These data correlate quite well with the differential potential of the MMTR OE/KD cells (Figure 1).

To delineate the discrepancy between the expression level of commitment genes and the MMTR/Dmap1 and Suz12 binding kinetics to the promoters, we employed ChIP-qPCR to other epigenetic regulators (p300 and Jmjd3) and the modified histones (H2AZ and H3.3) (Figure 6B). Histone acetyltransferase p300 and Tip60 (a key subunit of the Tip60-p400 complex) directly interact with each other, leading to the H3K27ac modification and recruiting modified histones to the promoter [55], whereas H3K27 demethylase Jmjd3 (also known as KDM6B) catalyzes the demethylation of H3K27me2/3 [56]. Both H3.3 and H2A.Z histone variants are deposited to the promoters and enhancers at transcriptionally active genes and coding regions of highly expressed genes [57]. We found that both histone modification factors (p300 and Jmjd3) showed decreased recruitment to the stemness gene promoters and higher binding to the developmental gene promoters in d3 mock mESCs when compared to those in d0 mock cells (Figure 6B). However, regardless of MMTR/Dmap1 OE or KD, they showed a tendency of higher binding to the stemness gene promoters at d0 cells and lower binding to the developmental gene promoters, although the corresponding levels were dependent on the promoter context (Figure 6B). In addition, histone variant protein (H2AZ, H3.3) binding was not different from that of p300 and Jmjd3, in general, with some exception for stemness gene *Klf4* and developmental Tead2 promoters (Figure 6B). Therefore, the binding affinities of histone modification factors (p300 and Jmjd3) and modified histones (H2AZ and H3.3) were in correlation with the expression of target genes (Figure 6A and Figure S6A,B) and the H3K27 modification status (Figure S6C,D) but not with the MMTR/Dmap1 expression levels. These data also suggest that Suz12 (or PRC2 complex) functions as an on–off switch, whereas MMTR/Dmap1 in the Tip60-p400 complex executes gene expression by an unknown mechanism via promoter context-dependent recruitment of other factors including Myc module factors during mESCs differentiation.

## 4. Discussion

The Myc targets are involved predominantly in cellular metabolism, cell cycle, and protein synthesis pathways, whereas the targets of core factors are more related to the development and transcription [8]. The core ESC factors select ESC genes through the recruitment of RNA Pol II, while c-Myc functions to control gene expression through the transcription pause [15]. Indeed, Myc-bound sites had shown higher levels of p-TEFb occupancy with increased levels of elongation-associated RNA Pol II phosphorylation [16]. Although approximately one-third of all active ESC genes are bound by both c-Myc and the core ESC pluripotency factors [14], previous studies proposed that targets occupied by the core pluripotency factors are different from genes bound by Myc [7,8]. In this study, almost all MMTR/Dmap1-bound regions showed a strong association with Max and Myc in addition to other Tip60-p400 complex proteins (Figure 3), and 34% (579/1698) of MMTR/Dmap1 target genes were co-occupied by Myc (Figure S5). These findings suggest that MMTR/Dmap1 functions as a member of the Myc module in the Tip60-p400 complex. Therefore, the Tip60-p400 complex recruited to the chromatin via binding to Myc module functions as a global transcription co-activator at most active Pol II promoters in ESCs.

The active stemness genes in ESCs must shut off during early differentiation commitment, while the lineage commitment genes must concurrently turn on. A previous study indicated that PcG proteins induce the ESCs to acquire and maintain specific development gene expression programs by forming a poised bivalent chromatin structure of the target gene promoters [45]. In this scenario, there is an antagonistic association between the Tip60-p400 complex and PcG proteins in general. However, a residual co-occupancy of both complex proteins is present in many cases. Therefore, our data are consistent with the hypothesis that dynamic interaction between Tip60-p400 and PcG proteins with the promoters of the mESC commitment genes exerts rapid cell fate decision. Importantly, H2A.Z showed strong association with the Tip60-p400 complex proteins in all ESC commitment genes, regardless of their expression changes (up- or downregulation) during differentiation (Figure 4). Since H2A.Z depletion caused expression of poised genes with concomitant loss of PRC components [58], it is plausible that H2A.Z association with Tip60-p400 is involved in the recruitment of PRCs.

In contrast to moderate or weak association between the PcG proteins and the Tip60-p400 complex in MMTR/Dmap1 downregulated genes, rapid transcription repression can be achieved by the strong association between PRCs and the Tip60-p400 complex. This leads to a transiently poised bivalent chromatin state. Likewise, dissociation of PRCs from the chromatin through an unknown mechanism releases genes from the transcriptional pausing. In ESCs, most bivalent promoters are thought to harbor a paused RNA pol II enzyme, although there is no evidence of this [59]. In most paused genes in ESCs, the Tip60-p400 complex significantly co-localizes with the H3K4me3 and acts to downregulate gene expression in ESCs [60]. Although there is no direct evidence connecting these two events (i.e., bivalent chromatin and transcriptional pausing), our previous finding that MMTR/Dmap1 acts as a corepressor to prevent RNAPII phosphorylation [28] supports the transcriptional pausing function of the Tip60-p400 complex in the bivalent gene promoters. Furthermore, Bmi1, a PRC1 protein, forms a ternary complex with MMTR/Dmap1 and Dnmt1 in association with gene silencing [61]. Interestingly, Chen and colleagues [62] showed that R-loops, RNA–DNA hybrids, modulate the binding of Tip60-p400 and PRC2 complexes in mESCs. PRC2 binds promiscuously to nascent RNA transcripts, and the level of RNA binding by the PRC2 catalytic subunit Ezh2 correlates with the RNA abundance [63]. The RNA binding by PRC2 inhibits its H3K27 methyltransferase activity [64]. In agreement with these findings, PRC2 components bind to both silent and active genes, and active genes bound by PRC2 are not marked by H3K37me3. Like PRC2, the Tip60-p400 histone acetyltransferase complex binds to nascent transcripts, although transcription promotes chromatin binding of Tip60-p400, but not PRC2 [62]. Therefore, a crosstalk between Tip60-p400 and PRC proteins might be important for balancing between the stemness and early lineage commitment, although the underlying mechanism should be further dissected.

MMTR/Dmap1 functions as a somatic Dnmt1- or HDAC2-mediated transcription repressor [17]. Homozygous MMTR/Dmap1 −/− embryos showed lethal phenotypes at the preimplantation stage due to the problems of methylation maintenance, and preimplantation epigenetic reprogramming [33]. Here, we found that MMTR/Dmap1 does not associate with Dnmt1-involved CpG methylation in the regulation of ESC commitment genes (Figure 3). This is conceivable, since the binding of PcG proteins in ESCs may repress genes during the later differentiation stages through more stable silencing mechanisms (DNA methylation) [65]. Furthermore, we previously reported that MMTR/Dmap1 is an intrinsic negative cell cycle regulator in somatic cells that modulates the CAK kinase activity via interaction with MAT1 [29]. Although this was not examined in this study, MMTR/Dmap1 may not serve as a cell cycle regulator. Recent findings showed that the pluripotency control in ESCs is hardwired to S and G2 phases, enacted by the ataxia telangiectasia mutated (ATM)/ATR-mediated replication checkpoint and Cyclin B1 pathways [66]. We showed that MMTR/Dmap1 plays a critical role in the regulation of commitment. However, it has a negligible effect on the mESCs self-renewal maintenance (Figure 1). These phenotypes in MMTR/Dmap1 KD and OE cells are very similar to those of PcG proteins [67]. PcG proteins are not necessary for the maintenance of self-renewal in ESCs, but rather they are thought to prepare ESCs for lineage commitment by the temporal control of the expressions of key developmental genes [67]. PRC2-deficient ESCs, as well as those lacking RING1B, fail to maintain properly the expression of lineage-specific genes [68]. Therefore, it is reasonable to suggest that the MMTR/Dmap1 is involved in the proper expression of stemness and developmental genes with Tip60-p400 complex by controlling the bivalent histone marks in their promoter regions via crosstalk with the PRC complexes. At this point, it is clear that additional analysis is needed for the identification and characterization of fraction(s) of MMTR/Dmap1 present in Tip60-p400 binding sites, since the related information would increase the depth of our findings. Exact binding site of Tip60-P400–MMTR/Dmap1 complex in regulatory regions could be identified by molecular and biochemical analyses, such as pull-down assay, but based on our results, we suggest that MMTR/Dmap1 binding regions have strong association with Tip60-p400. Further studies will provide valuable information about interaction between MMTR/Dmap1 and Tip60-p400 for regulation of gene expressions during maintenance and differentiation of mESCs.

Although the precise molecular mechanisms of PcG-mediated repression are still unknown, they might comprise two scenarios: (i) direct inhibition of the transcriptional machinery, PRC1-mediated ubiquitylation of lysine 119 of histone H2A, or (ii) chromatin compaction [69]. Recently, Tamburri et al. [70] revealed that the H2AK119ub1 deposition is essential for repression of the PcG target genes and H2AK119ub1 loss induced displacement of PRC2 activity and loss of H3K27me3 deposition in ESCs. In addition, PRC1 catalytic activity is crucial for the PcG-mediated gene repression by long-range chromatin interactions among PcG target sites [71]. Importantly, genes silenced by PcG proteins in ESCs maintain the potential to be activated at differentiation [72]. Furthermore, during cell-fate transitions, PcG proteins are re-recruited to distinct progenitor-specific targets and function as regulators of subsequent differentiation steps [73]. Regarding poorly understood reprogramming mechanisms involved in PRC and the related histone modification, Zheng et al., reported widespread resetting of epigenome plasticity in gametogenesis and early development by bivalent regulations of H3K27me3 [74]. In addition, Du et al. [75] recently revealed a unique PcG-regulated chromatin architecture with H3K27me3-marked PcG-associating domains in late-stage mouse oocytes. Therefore, PcG proteins serve as reversible repressors for genes involved in the lineage commitment. In more detail, PcG proteins can postpone fate decisions due to the fact of their bivalent domain activity and contribute to pluripotency.

The context-dependent and transient repression exerted by PcG proteins implies a dynamic control of PRC function during differentiation, possibly through the cooperation with auxiliary proteins such as sequence-specific transcription factors or enzymes modifying PRC core components. Chromodomain helicase DNA binding protein 4 (Chd4) in the Nucleosome Remodeling Deacetylase (NuRD) complex is essential for the localization of Ezh2 at the promoter of the key astrogenic marker gene *GFAP* (glial fibrillary acidic protein) and is required for its transcriptional suppression [76]. PcG proteins have been postulated to temporarily restrict gene expressions poised for activation during the lineage commitment [73]. DNA methylation, on the other hand, is generally regarded as a more permanent mechanism that might be incompatible with the rapid transcriptional activation. Therefore, it is intriguing to speculate that PcG-mediated silencing might replace more stable epigenetic mechanisms during cell-fate decision to induce gene activities specifying terminal differentiation. Chd4 is essential for Ezh2-mediated silencing of the *GFAP* promoter before the onset of astroglial differentiation [76]. It implies that chromatin remodeling might be necessary for the deposition of an alternative epigenetic mark. A recent study demonstrated that NuRD complex components are enriched at a subset of the PcG target genes, and NuRD-mediated deacetylation of histone H3K27 enables PRC2 recruitment and subsequent H3K27 trimethylation of NuRD target promoters [77]. NuRD has been shown to maintain the barrier between embryonic and trophoblast cell fates in ESCs [78]. Importantly, while ES cells lacking NuRD activity possess the ability to form trophoblast cells, in the absence of trophoblast-inducing external stimuli, they remain as self-renewing ESCs [78]. Furthermore, Kim et al. [37] recently reported that Mbd2–NuRD chromatin remodeling complex regulates erythropoiesis via interaction with CP2c complexes, and therefore, it is plausible that NuRD complex plays a crucial role in the cell-fate decision.

Promoters and enhancers control the temporal and spatial gene expressions, and approximately 70% of the most common promoters in the vertebrate genome are associated with the CpG islands (CGIs) [79,80]. About half of all CGIs contain transcriptional start sites (TSSs), and the other half, called orphan CGIs, are either within or between transcription units [81]. In addition, GC repeat motif are enriched in broadly active enhancers compared to both the genomic background and context-specific enhancers [82], and Steinhaus et al. [83] recently reported CGI-associated transcribed enhancers, which have pervasive and CpG-dependent promoter-like characteristics. In this study, we observed that GC-rich regions could serve as novel binding consensus sequences of MMTR/Dmap1 (Figure S5B–F). Furthermore, the majority of MMTR/Dmap1 binding regions in target genes (+/− 10 Kb from TTS) were at the promoter sites (39.6 or 41.6% in up- or down-target genes, respectively), and MMTR/Dmap1 was bound to other regions, including 5’UTR, exon, intron, and distal intergenic regions (Figure S5G). Considering these finding together with previous results, we speculate that MMTR/Dmap1 binds to GC-rich regions including common/orphan promoters, CGI-associated enhancers, and other putative regulatory regions of target genes to control their expressions. Therefore, further in-depth studies of the MMTR binding regions are needed, since they will broaden our understanding of the role of MMTR/Dmap1 in mESC dynamics.

Meanwhile, it is well known that the self-renewal mechanism of hESCs differs from that of the mESCs, although both of them are established from the inner cell mass of mouse or human preimplantation embryos. For example, hESCs respond to signaling between the NODAL/ACTIVIN-A-activated fibroblast growth factor (FGF) and transforming growth factor-beta (TGF-β) signaling pathway to sustain prolonged self-renewal, but different growth factors, leukemia inhibitory factor (LIF), and bone morphogenic protein 4 (BMP4) are required for mESCs [84]. To check the similarity of MMTR/Dmap1 function in hESCs, we analyzed the hESC GSE15257 microarray dataset to compare MMTR/Dmap1 target genes in mESCs. Although it did not correspond to d3 of mESCs, expression of hESC differentiation d12 was compared to MMTR/Dmap1 up- and down-target gene in mESCs (Figure S7). Among the 697 genes analyzed, 42% (292/697) of MMTR/Dmap1 target genes in hESCs were consistent with those of mESCs (Figure S7A) and GO analysis of these genes revealed several interesting pathways (e.g., cell proliferation, cell cycle, DNA damage response/repair, signaling pathway (Figure S7B). Here, we speculated that function of MMTR/Dmap1 in hESCs would be similar in mESCs and further studies will be required for its significance.

## 5. Conclusions

**Figure 7:**
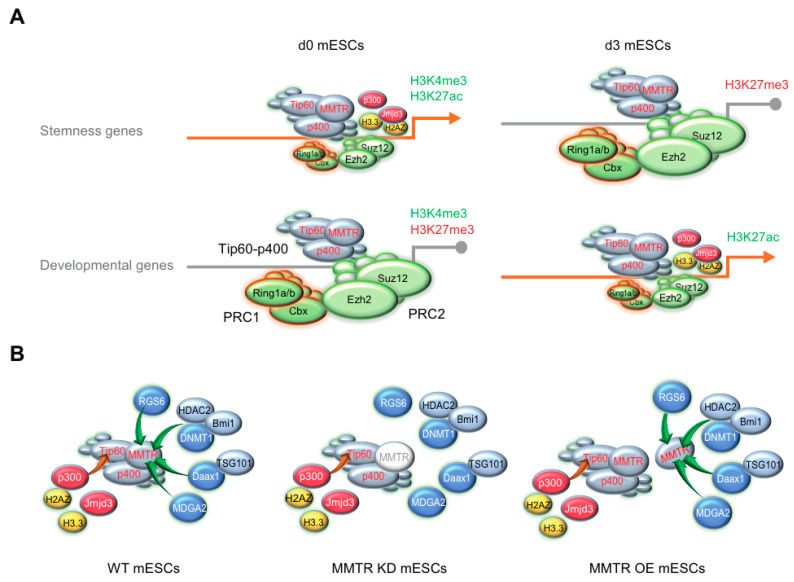
Schematic model of crosstalk among Tip60-p400 and PRC complexes (PRC1 and PRC2) on commitment genes expression. (**A**) PRC complexes function as an on–off switch of gene expression whereas the Tip60-p400 complex executes gene expression via recruiting other active histone modification factors and histones. The Tip60-p400 complex steadily stays on the promoter regions of both stemness and developmental genes regardless of differentiation status (d0 versus d3). On the contrary, binding affinity of PRC complexes determines whether target genes expression goes or not, such that strong binding of PRC complexes in stemness genes at d3 and developmental genes at d0 causes H3K27me3 modification leading gene expression shut down. Weak binding of PRC complexes in the promoters of the d0 stemness and d3 developmental genes allows H3K27ac modification and modified histone (H2AZ and H3.3) binding to the promoter by recruiting p300 and Jmjd3 via interaction with the Tip60-p400 complex. (**B**) Molecular model showing that arbitral downregulation or upregulation of the MMTR/Dmap1 expression leads upregulation of stemness genes at d0 mESCs. In normal WT mESCs where PRC complexes weakly recruited to the promoters, the Tip60-p400 complex could recruit p300 via interaction with Tip60. It subsequently leads H3K27ac modification in and modified histones binding to the promoter in a competitive manner with other inhibitory factors (RGS6, DNMT1-HDAC2-Bmi1, Daax1-TSG101, or MDGA2) recruited via interaction with MMTR /Dmap1 (left). The lack of MMTR/Dmap1 occupancy in Tip60-p400 by MMTR KD could not recruit those inhibitory factors due to the lack of their interacting partner MMTR, strengthening the p300 recruitment to the Tip60-p400 complex (middle). By MMTR OE, surplus MMTR outcompetes those inhibitory factors rendering higher interaction of p300 to Tip60 in the complex (right).

Here, we identified a novel mechanism of MMTR/Dmap1 in terms of bivalent regulations in maintenance and differentiation of mESCs. The Tip60-p400 complex, including MMTR/Dmap1, binds to almost all preferential genes for the maintenance of pluripotency. MMTR/Dmap1 regulates bivalent genes through intimate interaction with PRC complexes in mESCs (Figure 7). In normal WT mESCs where PRC complexes weakly recruited to the promoters, the Tip60-p400 complex could recruit p300 via interaction with Tip60 [55]. It subsequently leads to the H3K27ac modification and modified histones binding to the promoter in a competitive manner with other inhibitory factors, i.e., RGS6 [85], DNMT1-HDAC2-Bmi1 [61], Daax1-TSG101 [86], or MDGA2 [87] that are recruited via interaction with MMTR/Dmap1. The lack of MMTR/Dmap1 occupancy in Tip60-p400 by MMTR KD could not recruit those inhibitory factors due to the lack of their interacting partner MMTR, strengthening the p300 recruitment to the Tip60-p400 complex. By MMTR OE, surplus MMTR outcompetes those inhibitory factors rendering stronger interaction of p300 with Tip60 in the complex. In the present study, we propose that the multi-faceted regulator MMTR/Dmap1 is a molecular rheostat needed for the fate decision of the pluripotent embryonic stem cells. Understanding MMTR/Dmap1 molecular mechanisms by elaborative MMTR/Dmap1 modifications at transcriptional or translational levels will be a crucial next milestone.

## Supplementary Materials

The following are available online at https://www.mdpi.com/2073-4409/9/5/1190/s1, Figure S1: Establishment and characterization of MMTR mutant cell lines; Figure S2: The contribution of MMTR/Dmap1 mutant cells and their effects on the mESC differentiation; Figure S3: High throughput expression analysis of each MMTR/Dmap1 mutant cell by microarray; Figure S4: MMTR/Dmap1 does not regulate trophoblast marker genes; Figure S5: Tip60-p400 complex subunit MMTR/Dmap1 regulates the expression of genes involved in maintenance and differentiation of mESCs; Figure S6: Differentiation dependent expression changes of selected commitment genes and MMTR/Dmap1 dependent H3K27 modification status in their promoters; Figure S7: Comparison of MMTR/Dmap1 target genes in mouse and human ESCs; Table S1: List of primers for RT-PCR and ChIP-qPCR during differentiation of mESCs; Table S2: List of significantly up-regulated MMTR.Dmap1 target genes during differentiation of mESCs.
