# Supplementary material for: MMTR/Dmap1 Sets the Stage for Early Lineage Commitment of Embryonic Stem Cells by Crosstalk with PcG Proteins
Source: Cells. 2020 May 11;9(5):1190. doi: 10.3390/cells9051190 (PMC7290897; doi:10.3390/cells9051190)
Supplement: Supplementary file 1 [file cells-09-01190-s001.pdf]

cells

Supplementary information

Table S1. List of primers for RT-PCR and ChIP-qPCR during differentiation of mESCs.

Gene	Forward (5' to 3')	Reverse (5′ to 3')	Amplicon size
RT-qPCR			
Brachyury	CCAGCTCTAAGGAACCACCG	ACTCCGAGGCTAGACCAGTT	137 bp
Cdx2	TGTACACAGACCATCAGCGG	CCAAATTTTAACCTGCCTCTCGG	125 bp
Dnmt3b	GCCAGACCTTGGAAACCTCA	TTGTTTCCTGAAAGAAGGCCC	139 bp
Dppa5a	GACCCTCGTGACCCGTAAAG	CCCTGTGGGCCAAACAGATA	117 bp
Enpp2	CAGAGGAAGTCAGCAGACCC	CCAATTTGTTCTTTGGCTCTACCT	105 bp
Eomes	ACAACTACGATTCATCCCATCAGA	GGGCTTGAGGCAAAGTGTTG	96 bp
Esrp1	GGCCTGGCCTACAATACTGG	CATCCTCGGTTGCATACTGGT	73 bp
Esrrb	CCTTAGTCACAAAGAGACACAGAC	TTCGGTTCAGCAGCATGGTT	150 bp
Gapdh	CGTGCCGCCTGGAGAAACC	TGGAAGAGTGGGAGTTGCTGTTG	144 bp
GATA4	CCCTGGAAGACACCCCAATC	ATTGCACAGGTAGTGTCCCG	127 bp
Grb10	GCGTCCTAGCTCTGTACCTTG	GACCATGCTCTGGTTGGCTT	109 bp
Hand1	CGGAAAAGGGAGTTGCCTCA	GGTGCGCCCTTTAATCCTCT	81 bp
Kdr	GAGATTACAAGGCTTTCAGCA	CCTCCGTTTGAGATGAGAGAG	119 bp
Klf4	GACTAACCGTTGGCGTGAGG	CGGGTTGTTACTGCTGCAAG	106 bp
Lefty1	CTCGATCAACCGCCAGTCCT	TGCCACCTCTCGAAGGTTCTG	150 bp
Lefty2	CCTGGACAGCGCGGATG	GCCTGCCACCTCTCGAAAAT	136 bp
Lrp2	AGCGGCTACAGTGGAGAGTA	AAGAGCCATTGTCGTCCCTG	72 bp
Nanog	AGGATGAAGTGCAAGCGGTG	CTGGTGCTGAGCCCTGAAT	76 bp
Nestin	GAGGCGCTGGAACAGAGATT	TAGACCCTGCTTCTCCTGCT	105 bp
Nodal	CTGGCGTACATGTTGAGCCT	GGTCACGTCCACATCTTGCG	87 bp
Pdgfa	AGGAGGAGACAGATGTGAGGT	TTCAGGAATGTCACACGCCA	81 bp
Pdha2	TGGAATGGGAACCTCCAACG	CTGCAAACTTGGTTGCCTCC	128 bp
Pou3f1	TCCCCACAGCGAAAGGTAAC	GGGGACTCTCCTCTCTTCGT	74 bp
Pou5f1 (Oct4)	GCAGATAGGAACTTGCTGGGT	CACCTTTCCAAAGAGAACGCC	149 bp
Slc27a2	CATCGTGGTTGGGGCTACTT	GGTACCGAAGCAGTTCACCA	119 bp
Sox17	TTCTGTACACTTTAATGAGGCTGTTC	TTGTGGGAAGTGGGATCAAG	113 bp
Tead2	TTTTCCCAAACAAAGCTCCCG	ATCTGGGCAAACCCCAAGCA	128 bp
Tfap2c	CACGGGTAACCGGACTGTG	GAGTCGTGGCGATCCTGAG	114 bp
Tfcp2l1 (CRTR)	AGAAGGGAGTGCCTTTTCGG	CCCTTGGGCTTGAACACCTT	115 bp
Trio	CCGACGGGACTCTCAAGAAA	TTCTCGTGGCTTCCTGTACC	128 bp
Zfp42 (Rex1)	CTGGGTACGAGTGGCAGTTT	CCATTTCTCTAATGCCCACAGC	98 bp
ChIP-qPCR			
Dnmt3b	GGGTTAAGCGGCCCAAGTAA	GCGCTCACCTGTCGTGA	140 bp
Klf4	CCTCTCTCGCATACGCACG	CCCTTCTCCTAGCTTCTGAGAT	146 bp

Lefty1	GCATCCAGCAGAGAACGTGA	AGGACATTCAGGACAGGGGT	148 bp
Nanog	ACAATGTCCATGGTGGACCC	ACCCTACCCACCCCCTATTC	106 bp
Pdha2	TGGAATGGGAACCTCCAACG	CTGCAAACTTGGTTGCCTCC	128 bp
Pou3f1	AAGCCGATCCGAATCTGTCC	TCTAGACTCAGCGCCACAAC	78 bp
Pou5f1 (Oct4)	TGGGAGGTGAGCATGACAGA	CACATGTGTGGAAGTCCCCT	128 bp
Tead2	ACTGAGTCCCAAGGCCTGAA	AAAAGGGGGCATAGTCAGGG	98 bp
Tfcp2l1 (CRTR)	AGCCCGAACACTACAACCAG	TGGAGGCCTGAGGTTAGTCA	105 bp

Table S2. List of significantly up-regulated MMTR/Dmap1 target genes during differentiation of mESCs. Genes were categorized according to GO groups.

Term group	Target gene #	Occupancy	Gene list
Embryonic morphogenesis	96	11.20%	Aatk, Acd, Acsl4, Acvr2b, Add1, Ahi1, Bcl2l11, Bcl9l, Bcor, Bmpr1a, Bmpr2, Bnip2, Cebpb, Col2a1, Cthrc1, Cul4a, Cul7, Dab2, Dicer1, Dlg3, Dlg5, Dpysl2, Dusp4, Dusp6, Efna5, Ell3, Epb4.1l5, Ercc2, Ets2, Etv2, Fzd7, G2e3, Gdf1, Gja1, Glipr2, Gnaq, Gnas, Gpc3, Gsk3b, Has2, Hectd1, Hey1, Hook3, Hs2st1, Ilk, Kdm2b, Klf10, Krt8, Llgl1, Lrig3, Lrp2, Man2a1, Med12, Megf8, Mfsd7b, Miat, Mtcl1, Myh10, Myo6, Nsdhl, Otx2, Pbx1, Prickle1, Prkaca, Prkacb, Prkra, Pygo2, Sall2, Sema3f, Skil, Slc40a1, Slc9a3r1, Smad1, Smad3, Smo, Smo., Sox11, Sox4, Spg20, Srsf1, Ssbp3, Stag2, Sulf1, Tab1, Tcf3, Tcf7, Tead2, Twsg1, Twsg1., Ube2b, Vangl2, Vangl2., Yap1, Yap1., Zbtb18, Zic2
Cellular differentiation	158	18.40%	Aatk, Acsl6, Acvr2b, Add1, Ahi1, Als2, Apbb1, App, Arhgef2, Atp6ap1, Bcl11b, Bcl9l, Bex2, Bhlhb9, Bmpr1a, Bmpr2, Bnip2, C1galt1, Carm1, Cbfb, Cd276, Cebpb, Cebpg, Chrnb1, Clic1, Clic4, Cthrc1, Cul4a, Cul7, Cxadr, Dab2, Dicer1, Dlg5, Dnmt3b, Dpysl2, Dpysl5, Dusp6, Efna5, Ell3, Epb4.1l5, Erbb2, Ercc2, Errfi1, Etv2, Fam213a, Farp1, Fbln1, Fbxo45, Fdps, Fem1b, Fzd7, Gabrb3, Gak, Gja1, Glipr2, Gnaq, Gnas, Gprc5b, Gsk3b, Hacd1, Has2, Hey1, Hist1h4a, Hnrnpc, Homer1, Hook3, Igf1r, Ilk, Inppl1, Irf1, Itgb1bp1, Jmjd6, Kank1, Klf10, Klf13, Klf7, Kmt2e, Kras, Krt8, Lats1, Llgl1, Lrp8, Man2a1, Map2, Map3k1, Mapk12, Mapk8ip2, Med12, Megf8, Mfsd7b, Miat, Mllt4, Mycl, Myh10, Myo6, Myrf, Ndfip1, Ndn, Neu1, Nfatc4, Nlgn2, Nrep, Ntrk2, Numb, Olfm1, Otx2, Pacsin1, Palm, Pbx1, Pou3f1, Ppp1r9a, Ppp2r5d, Ppp3ca, Prickle1, Prkaca, Prkcz, Pten, Ptprs, Rab25, Ramp2, Rassf2, Rcor1, Sdc2, Sema3f, Sema6a, Skil, Slc9a3r1, Slitrk5, Smad1, Smad3, Smarca2, Smo, Sort1, Sox11, Sox4, Spg20, St6gal1, Stag2, Stau2, Stxbp1, Stxbp5, Tcf3, Tead2, Thy1, Tmem14c, Trim32, Triobp, Tspo, Ttc3, Twsg1, Unc5b, Vamp3, Vangl2, Vezf1, Yap1, Zfhx3, Zic2, Zswim6
Cell cycle	60	7.00%	Apbb1, App, Arhgef2, Bach1, Bcl2l11, Bhlhe40, Cables1, Calm2, Calm3, Ccar2, Cdc7, Csnk2a2, Ctdsp2, Cul4a, Cul7, Dgkz, E2f6, Eml1, Ercc2, Fem1b, Fign, Gja1, Hacd1, Igf1r, Ilk, Insr, Irf1, Klhl21, Kmt2e, Lats1, Lzts2, Map3k1, Mapk12, Mapre2, Mlxipl, Myh10, Nabp2, Nedd1, Nudt16, Pbx1, Pim2, Pkia, Ppp3ca, Prkaca, Prkacb, Prmt2, Pten, Rassf1, Skil, Slc9a3r1, Smad3, Sox4, Stag2, Tcf3, Trim32, Triobp, Ube2b, Uhrf2, Usp47, Wapal
Chromatin remodeling	48	5.60%	Aebp2, Apbb1, Arid1b, Auts2, Bahcc1, Bahd1, Bcor, Brd3, Carm1, Cebpg, Dnmt3b, Ehmt1, Gja1, Gnas, Gsk3b, Hist1h2bj, Hist1h3e, Hist1h4a, Hist2h2be, Hmgn3, Hnrnpc, Ing2, Jmjd6, Kansl3, Kdm2b, Kdm7a, Kmt2e, Mbd2, Nap1l1, Prkcb, Prmt2, Pygo2, Rcor1, Ring1, Satb1, Smarca2, Smarcd2, Smarce1, Snca, Suds3, Supt3, Tada1, Tcf3, Tdrkh, Ube2b, Uhrf2, Yeats2, Zmynd11

Cell Damage/Repair	52	6.10%	2810417H13Rik, Apbb1, Bmpr1a, Casp9, Ccar2, Cdc14b, Cebpg, Cops5, Csnk2a2, Cul4a, Dab2, Dgkz, Ell3, Ercc2, Fem1b, Fign, Get4, Gja1, Gstp1, Herpud1, Igf1r, Map3k1, Mapk8ip2, Nck2, Nfatc4, Nudt16, Pea15a, Pmaip1, Pten, Ralb, Rassf2, Rhno1, Sash1, Sh3rf1, Skil, Snca, Sox4, Spop, Stk32a, Supt3, Tbc1d5, Tgfbrap1, Traf4, Trim32, Trove2, Trp53bp1, Ube2b, Ube4b, Usp47, Vangl2, Xpa, Zmynd11
Apoptosis/Cell death	123	14.30%	Aatk, Ahi1, Apbb1, App, Bag3, Bcl11b, Bcl2l11, Bcl7c, Bex2, Bhlhb9, Bmpr2, Bnip2, Bok, Cadm1, Capn10, Casp9, Ccar2, Cebpb, Clic4, Col2a1, Csnk2a2, Cul7, Dab2, Ddit4, Degs1, Dhcr24, Dicer1, Dido1, Dusp6, Dyrk2, Egln3, Ell3, Epb4.1l3, Erbb2, Ercc2, Fbxo10, Fem1b, G2e3, Gabrb3, Gdf1, Gja1, Gnaq, Gsk3b, Gstp1, Herpud1, Hif3a, Hspd1, Igf1r, Ilk, Ing2, Irf1, Irs2, Ivns1abp, Kdm2b, Kras, Krt18, Krt8, Lzts2, Map3k1, Mdk, Mtch1, Nck2, Ncl, Nes, Nfatc4, Nme3, Nrbp2, Nsmaf, Ntrk2, Nuak2, Pacs2, Pea15a, Pim2, Plagl2, Plcg2, Pmaip1, Ppp2cb, Prkcb, Prkcz, Prkra, Prmt2, Pten, Ralb, Ramp2, Rassf2, Rhot2, Rnf130, Rtn3, Sema6a, Sh3rf1, Skil, Slc40a1, Slc9a3r1, Smad3, Smo, Snca, Sort1, Sox11, Sox4, Spop, St6gal1, Stk17b, Stk40, Stxbp1, Suds3, Sulf1, Tcf7, Tead2, Tgfbrap1, Tmem132a, Traf4, Trim32, Tspo, Ube2b, Ube2k, Ube4b, Unc5b, Usp47, Vegfb, Xpa, Yap1, Ypel3, Zmynd11

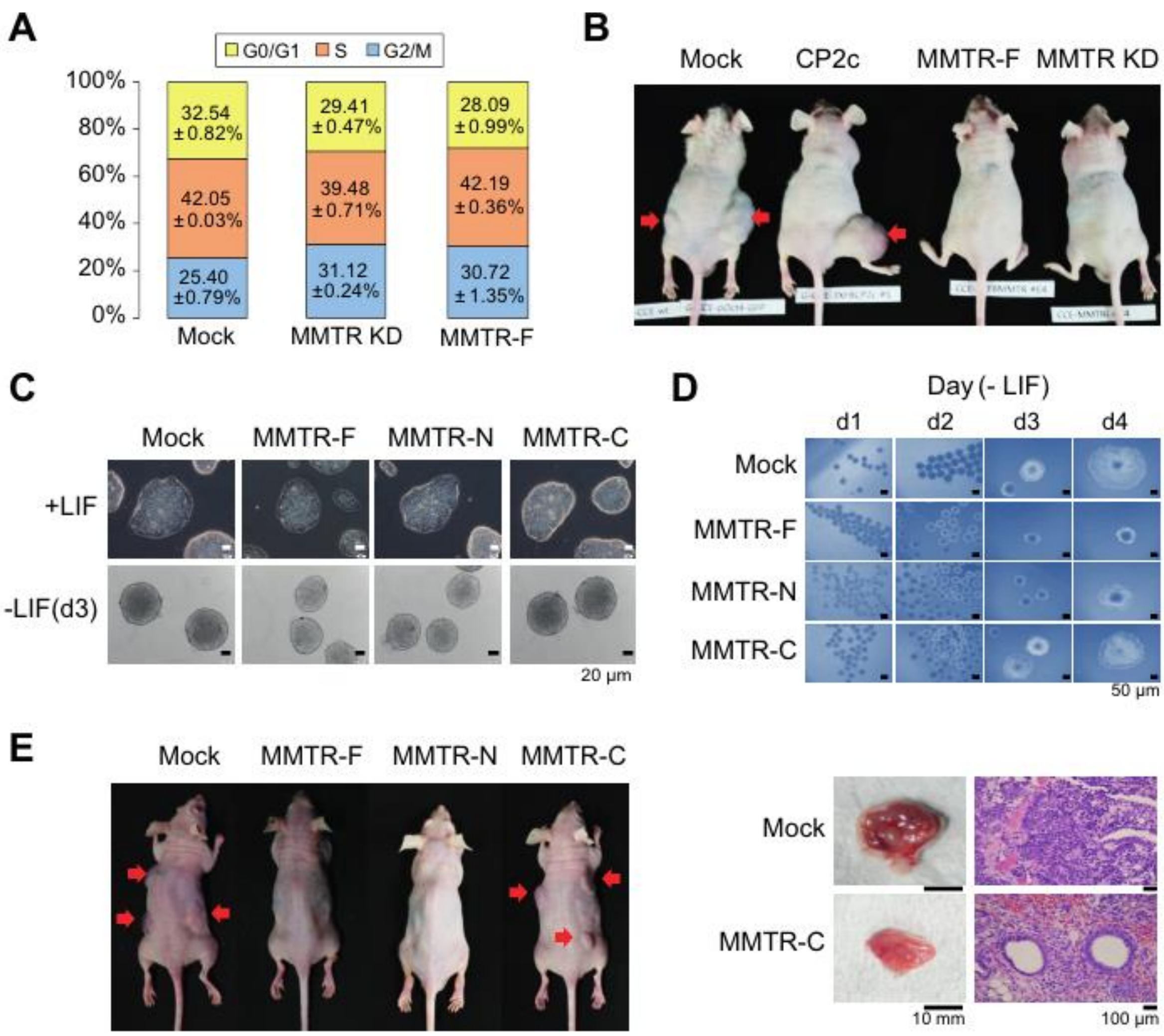

Figure S1. Establishment and characterization of MMTR mutant cell lines. (A-B) Establishment and characterization of MMTR KD and MMTR–F mESC lines. (A) Cell cycle profiles of cells cultured under LIF were analyzed by flow cytometry. (B) *In vivo* teratoma forming assays. Wild type and oncogenic CP2c-OE mESCs were as positive controls. (C-E) Establishment and characterization of MMTR-F, -N, and -C mESC lines. (C) Morphological images of colonies under phase contrast microscope and (D) EB images (embryoid body) for 4 days culture without LIF. (E) *In vivo* teratoma forming assays in mock, MMTR-F, -N and -C mESCs-injected mice (left) and teratoma lesions in mock and MMTR-C mESC injected mice (right).

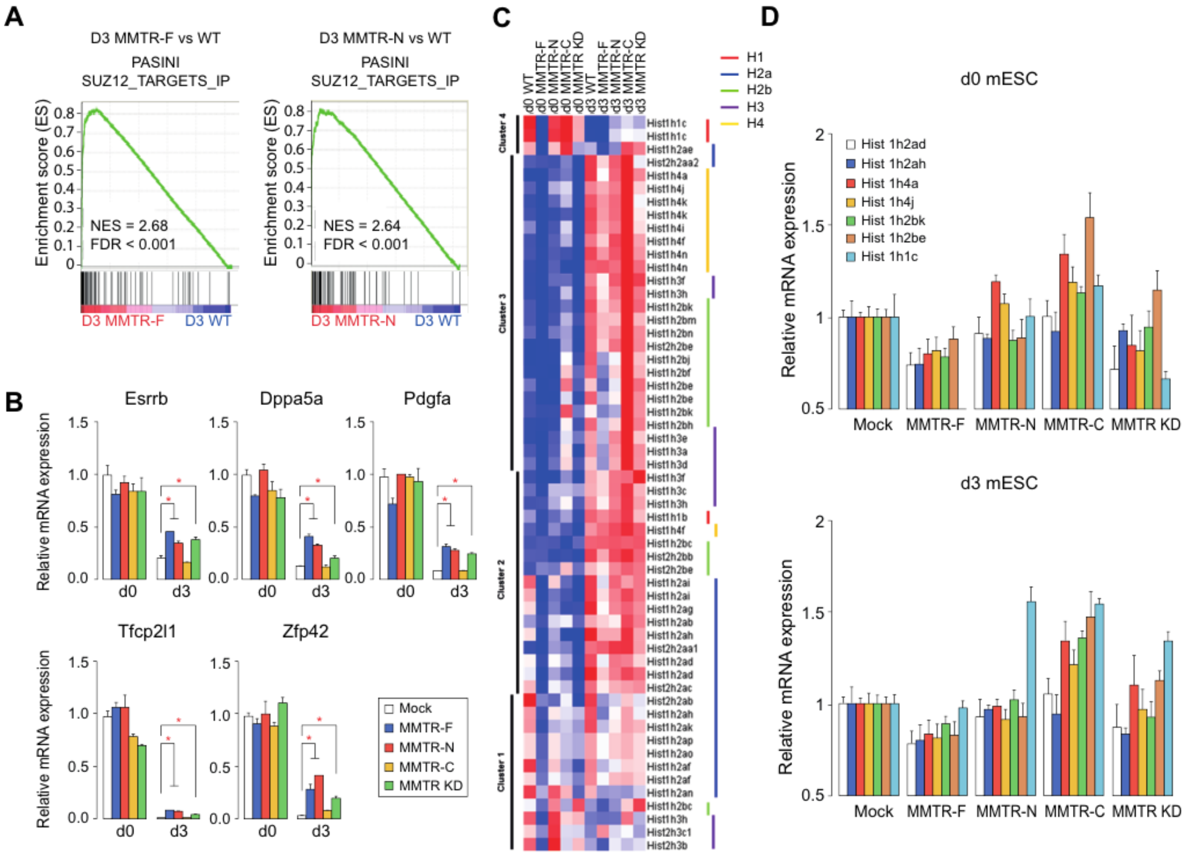

Figure S2. The contribution of MMTR/Dmap1 mutant cells and their effects on the mESC differentiation. **(A)** GSEA2 analysis of MMTR/Dmap1-full length or –N term overexpressed mESCs compared to wild type. **(B)** RT-qPCR of selected target genes from each mutant cell line under undifferentiation (d0) and differentiation condition (d3). **(C)** Heatmap showing expression levels of the histone gene cluster genes by microarray and **(D)** RT-qPCR showing expression levels of the selected histone genes in each MMTR/Dmap1 mutant at d0 and d3. n = 2. *, $p < 0.05$.

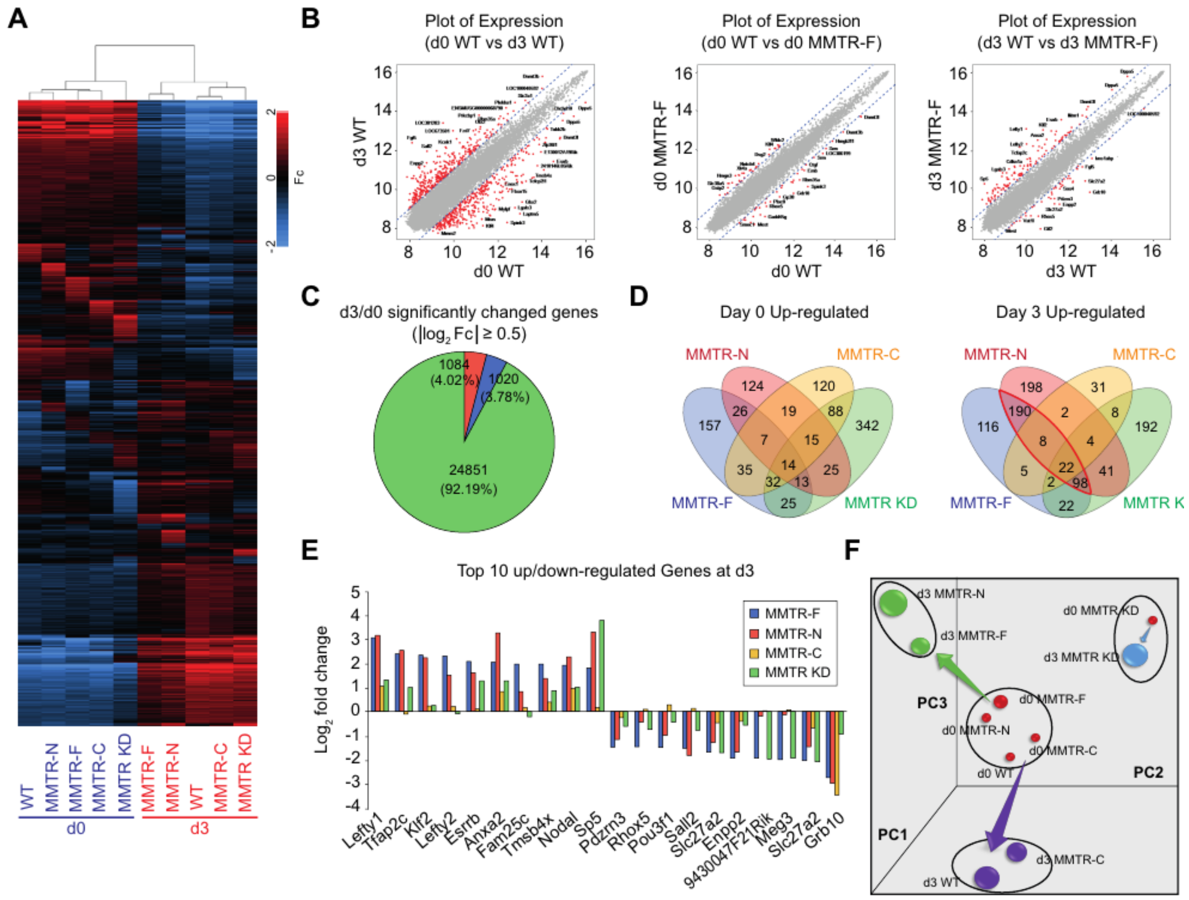

Figure S3. High throughput expression analysis of each MMTR/Dmap1 mutant cell by microarray. **(A)** Heat map analysis of each MMTR/Dmap1 mutant cell line on day 0 (d0) and day 3 (d3), and **(B)** scatter plots of datasets in wild type and MMTR/Dmap1-full length overexpressed mESCs on d0 and d3. Number of **(C)** preferentially expressed genes in wild type mESCs and **(D)** up-regulated genes in each MMTR/Dmap1 mutant cell for 3 days spontaneous differentiation. **(E)** RT-qPCR showing expression of selected up- or down-regulated genes in each MMTR/Dmap1 mutant cell line compared to wild type mESCs on d3. **(F)** Principal component analysis of gene expression in each MMTR/Dmap1 mutant cell.

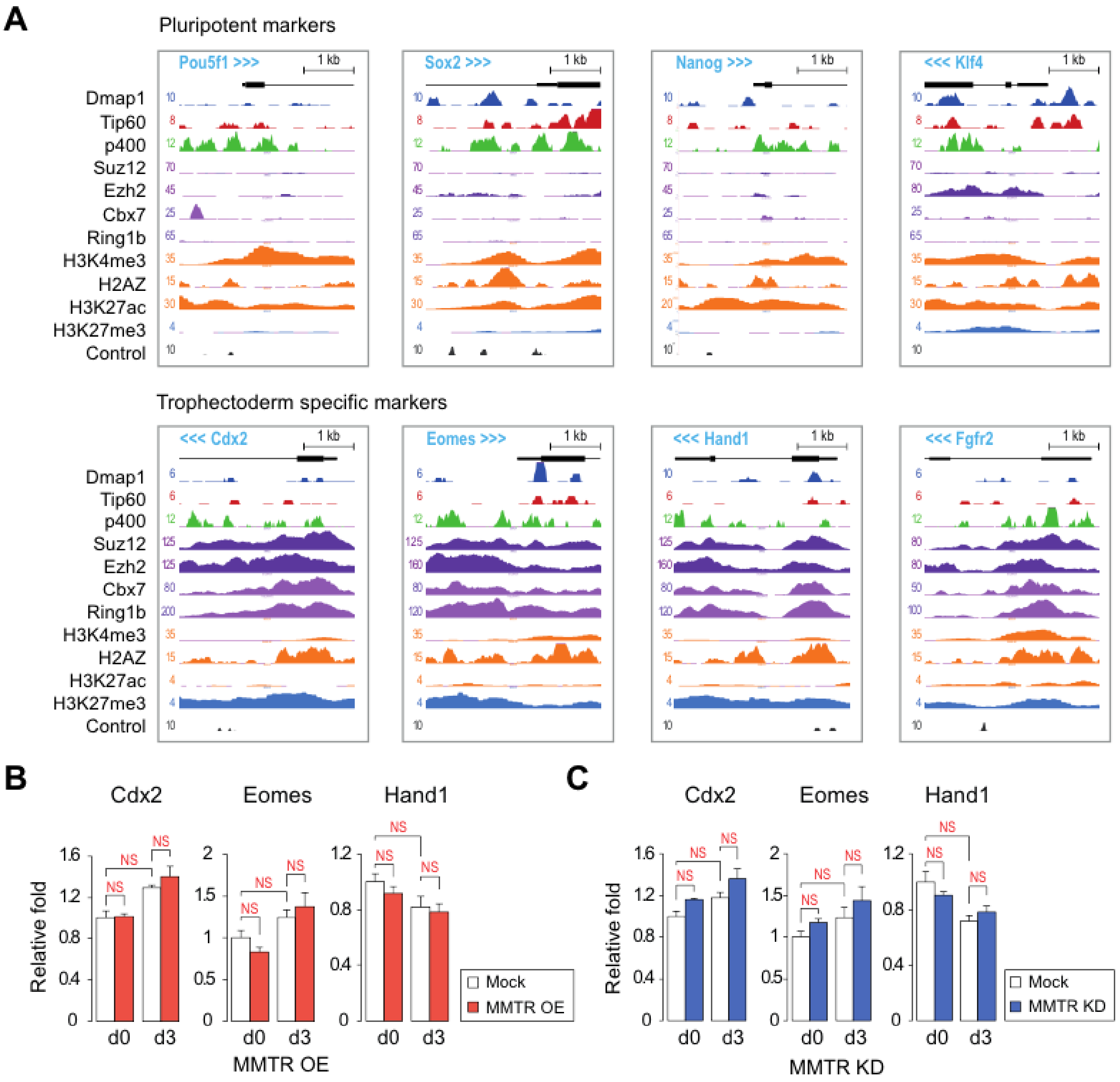

Figure S4. MMTR/Dmap1 does not regulate trophoblast marker genes. **(A)** Binding patterns of MMTR/Dmap1, Tip60-p400 complex, PRC1/2 complex, and H2A.Z, and activation or inactivation histone marks at regulatory regions including CpG islands of pluripotency or trophectoderm marker genes. **(B-C)** Trophectoderm marker gene expression profiles during maintenance or differentiation of MMTR/Dmap1-KD / OE cells by RT-qPCR. NS, no significant.

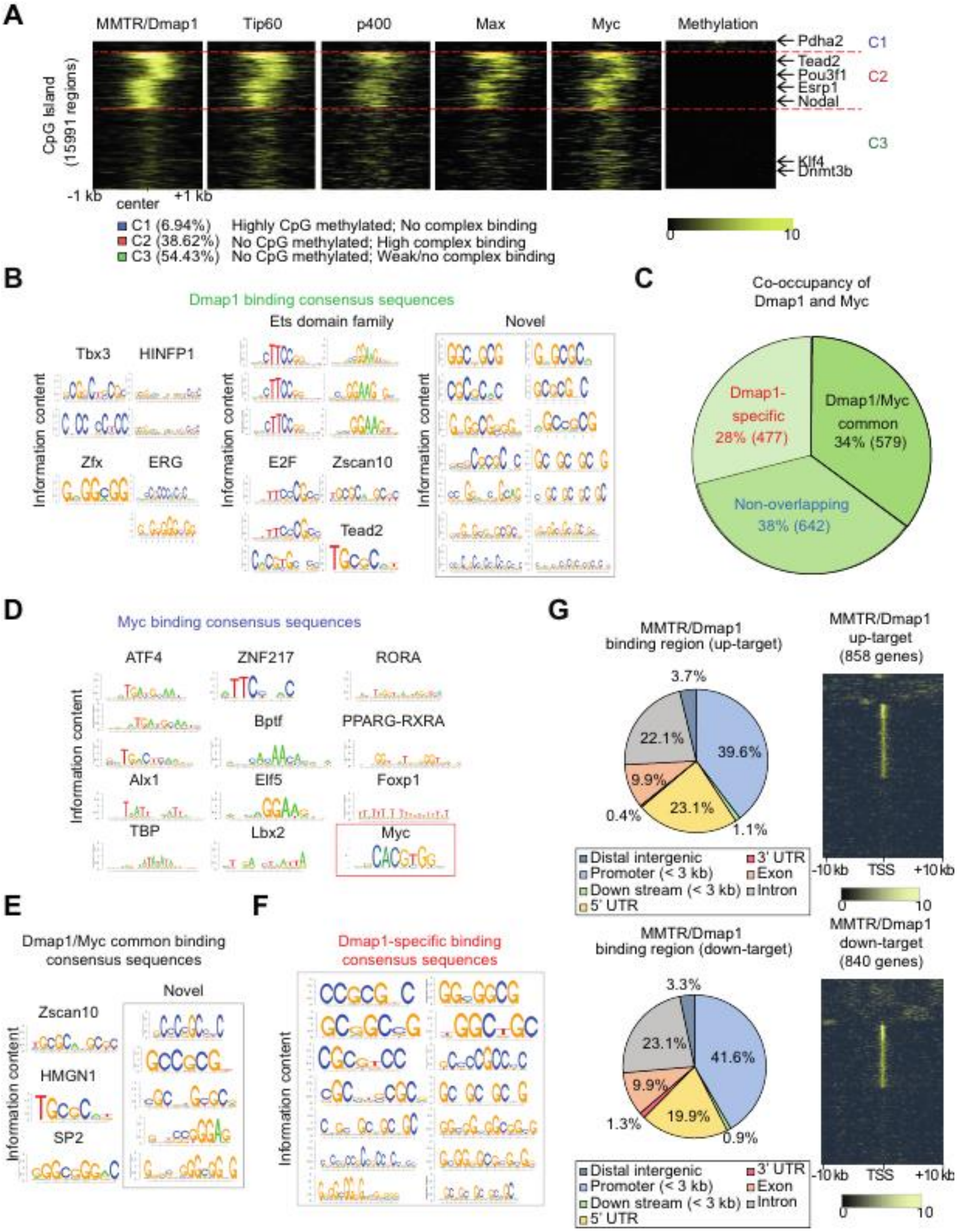

Figure S5. Tip60-p400 complex subunit MMTR/Dmap1 regulates the expression of genes involved in maintenance and differentiation of mESCs. (A) ChIP-seq heat maps of MMTR/Dmap1, Tip60, p400, Max, and Myc enrichment on the 15,991 CpG island-containing regions from –1 Kb to +1 Kb from each peak center. Heat maps are sorted by DNA methylation propensity. C1 to C3 regions represent three criteria. C1: high methylation, but no MMTR/DMAP1-Tip60-p400 complex binding (Pdha2). C2: No methylation, but high MMTR/DMAP1-Tip60-p400 complex binding (Tead2, Pou3f1, Esrp1, and Nadal). C3: No methylation as well as weak (or no) MMTR/DMAP1-Tip60-p400 complex binding (Klf4 and Dnmt3b). (B) Binding consensus sequences of MMTR/Dmap1 in TSS (-1 kb ~ +1 kb) of its 1698 target genes. Ets domain family and GC-rich sequences are enriched in MMTR/Dmap1 target genes. Tbx3, T-box transcription factor 3; HINFP1, Histone H4 transcription factor 4; Zfx, Zinc finger

protein X-linked; ERG, ETS transcription factor; Zscan10, Zinc finger and SCAN domain containing 10. (C) Co-occupancy with Myc in MMTR/Dmap1 target genes. Among 1698 target genes of MMTR/Dmap1, 579 genes (34%) are overlapped with those of Myc, and 477 genes (28%) are only MMTR/Dmap1-specific. In addition, both MMTR/Dmap1 and Myc bind to different regions of 642 genes (38%). (D) Consencus sequences of Myc binding in MMTR/Dmap1 target genes from panel (C). Myc consensus sequence is indicated by red square. ATF4, Activating transcription factor 4; ZNF217, Zinc finger protein 217; RORA, RAR related orphan receptor A; Alx1, ALX homeobox 1; Bptf, Bromodomain PHD finger transcription factor; PPARG-RXRA, Peroxisome proliferator activated receptor gamma-Retinoid X receptor alpha; Elf5, E74 like ETS transcription factor 5; Foxp1, Forkhead box P1; TBP, TATA-box binding protein; Lbx2, Ladybird homeobox 2. (E) Consencus sequences of MMTR/Dmap1 and Myc co-occupancy regions from panel (C). (F) Consensus sequences of MMTRDmap1-specific binding regions from panel (C). SP2, Sp2 transcription factor. (G) Distribution of MMTR/Dmap1 binding sites in the significantly up- (858, upper) and down-regulated (840, lower) gene regions (right). ChIP-seq heat maps (next to corresponding percentage distribution circle) are extending from –10 kb to +10 kb from each TSS. Each row represents a gene, and enrichment denoted in yellow. The colar bars indicate binding strength.

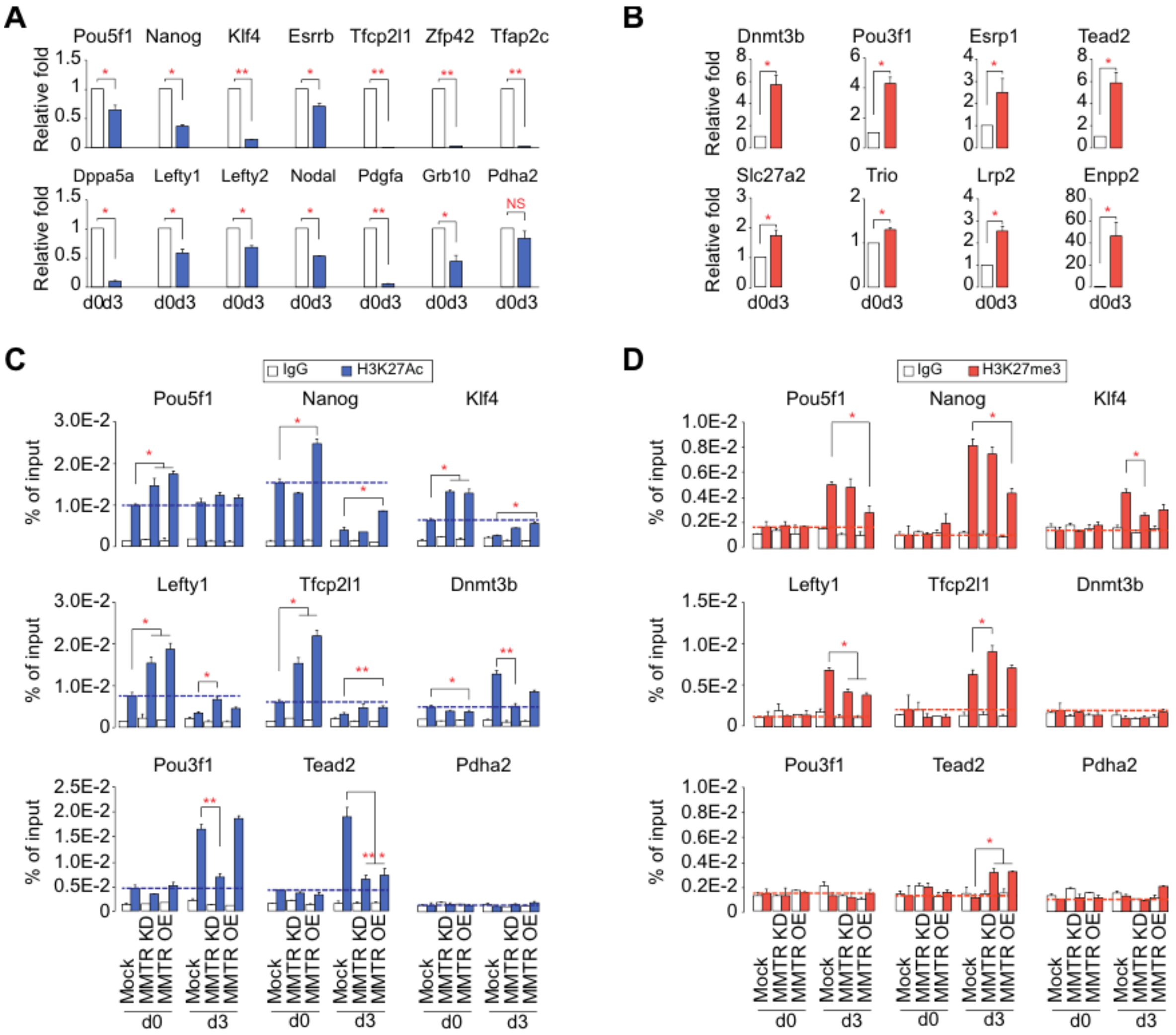

Figure S6. Differentiation dependent expression changes of selected commitment genes and MMTR/Dmap1 dependent H3K27 modification status in their promoters. (**A**,**B**) RT-qPCR graphs showing gene expression fold changes in MMTR/Dmap1 target genes (stemness genes; *A*, Developmental genes; *B*) of Figure 5A. (**C**,**D**) ChIP-qPCR graphs showing active (H3K27ac, *C*) and inactive (H3K27me3, *D*) marks at selected MMTR/Dmap1 target gene promoters in MMTR KD and OE cells of undifferentiated (d0) and differentiated (d3) mESCs. Pdha2 are used as a negative control and output values are represented as a percentage of the respective input DNA. *, $p < 0.05$; **, $p < 0.01$; NS, no significant.

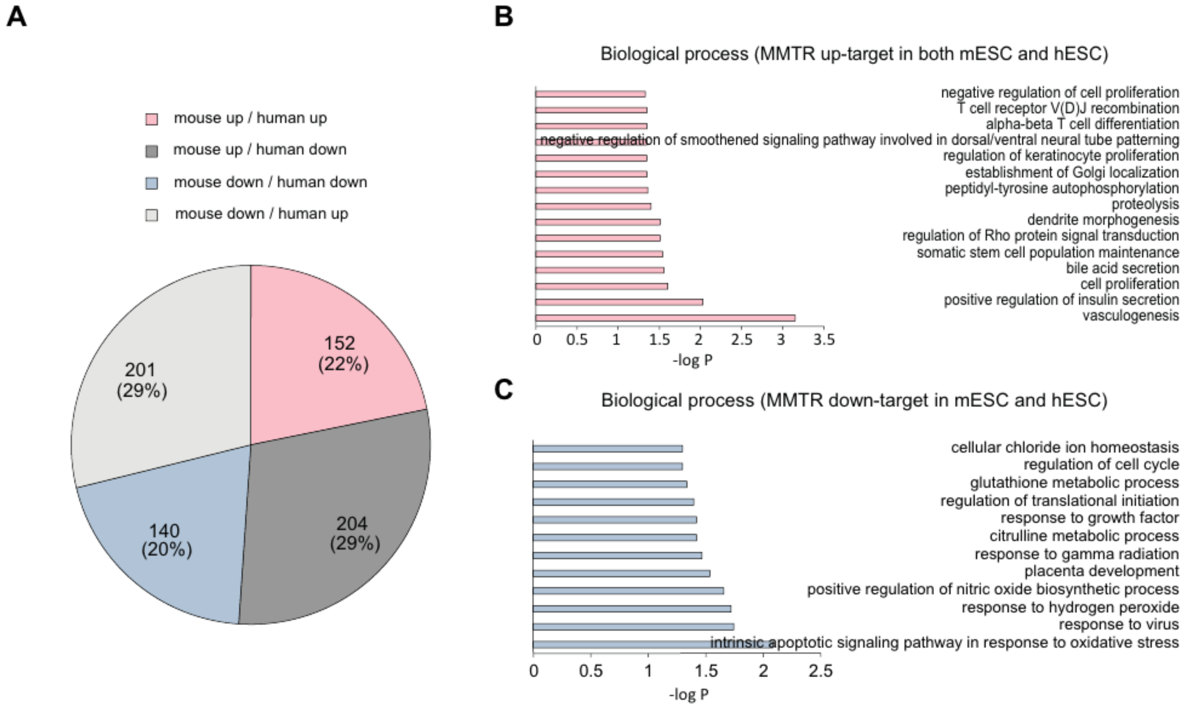

Figure S7. Comparison of MMTR/Dmap1 target genes in mouse and human ESCs. **(A)** The RNA expression profile change of hESCs differentiation (d0 and d12) (GSE15257) are compared to those of mESCs. GO term analysis of **(B)** up- or **(C)** down-regulated target genes in both mouse and human ESCs.
